# Methodological Quality and Risk of Bias Assessment of Cardiovascular Disease Research: Analysis of Randomized Controlled Trials Published in 2017

**DOI:** 10.3389/fcvm.2022.830070

**Published:** 2022-03-17

**Authors:** Odgerel Baasan, Omar Freihat, David U. Nagy, Szimonetta Lohner

**Affiliations:** ^1^Doctoral School of Health Sciences, University of Pécs, Pécs, Hungary; ^2^Cochrane Hungary, Clinical Centre of the University of Pécs, Medical School, University of Pécs, Pécs, Hungary; ^3^Institute of Geobotany/Plant Ecology, Martin-Luther-University, Halle, Germany; ^4^Department of Public Health Medicine, Medical School, University of Pécs, Pécs, Hungary

**Keywords:** randomized controlled trials, risk of bias, cardiovascular diseases, funding source, data monitoring committee, trial registration

## Abstract

**Background:**

All randomized-controlled trials (RCTs) are required to follow high methodological standards. In this study, we aimed to assess the methodological quality of published cardiovascular clinical research trials in a representative sample of RCTs published in 2017.

**Methods:**

Cochrane Central Register of Controlled Trials was used to identify cardiovascular clinical research trials with adult participants published in 2017. Overall, 250 (10%) RCTs were randomly selected from a total of 2,419 studies. Data on general trial characteristics were extracted and the risk of bias (RoB) was determined.

**Results:**

Overall, 86% of RCTs have reported at least one statistically significant result, with the primary outcome significant in 69%, treatment favored in 55%, and adverse events reported in 68%. Less than one-third (29%) of trials were overall low RoB, while the other two-thirds were rated unclear (40%) or with high RoB (31%). Sequence generation, allocation concealment, and selective reporting were the domains most often rated with high RoB. Drug trials were more likely to have low RoB than non-drug trials. Significant differences were found in RoB for the allocation concealment and blinding of participants and personnel between industry-funded and non-industry-funded trials, with industry-funded trials more often rated at low RoB.

**Conclusion:**

Almost two-thirds of RCTs in the field of cardiovascular disease (CVD) research, were at high or unclear RoB, indicating a need for more rigorous trial planning and conduct. Prospective trial registration is a factor predicting a lower risk of bias.

## Introduction

Randomized controlled trials (RCTs) are widely recognized as the most optimal methodology for causal inference, where humans are prospectively included and randomly allocated to groups to evaluate the efficacy and safety of an intervention (1). The strength of the RCTs comes from the randomization procedure, which ensures that all participants have the same chance of being assigned to each of the study groups (1) and guarantees that the characteristics of the participant are similar through the different groups at the baseline (2). However, the extent to which we can draw final conclusions based on RCTs strongly depends on how rigorous study methodology is; methodological inaccuracies during trial planning and conduct will subsequently reduce the reliability of results and their usability in medical practice (3). Biased results can finally lead to the underestimation or overestimation of the true intervention effect (3).

In cardiovascular disease (CVD) research, RCTs have been widely used to provide reliable knowledge on the best treatment strategies, such as therapies which are able to improve patient’s symptoms, correct disease markers, and improve clinical outcomes (4, 5). However, the risk of bias (RoB) has not been assessed in these studies.

The risk of bias (RoB) reflects the degree to which the results of a trial should be believed (6, 7). To reduce the possibility of RoB in RCT’s, the Cochrane Collaboration introduced a tool designed to appraise RoB (8, 9), involving six domains related to the internal validity of a trial: sequence generation, allocation concealment, blinding, incomplete outcome data, selective outcome reporting, and “other” potential threats to validity (6, 7). The risk of bias assessment enables the assessment of flaws in the trial design, conduct, and analysis that may affect study results (10).

In this study, we aimed to describe the reliability of evidence of cardiovascular diseases from a representative sample of cardiovascular RCTs published in 2017. Specific objectives were to examine: (1) the reliability of published cardiovascular trials using the RoB tool; (2) specific trial characteristics which increase the likelihood of unclear/high RoB; and (3) any potential differences in methodological issues between studies funded by the industry or the academy.

## Materials and Methods

### Sample Selection

We used the Cochrane CENTRAL Register of Controlled Trials to search for RCTs published in 2017 using subject headings and keywords related to adults (aged ≥ 18 years) and CVDs (such as, atherosclerosis, arrhythmia, cardiomyopathy, heart failure, hypertension, ischemic heart disease, heart attack, angina, sudden death, cardiac arrest, hypercholesterolemia, high blood pressure, CVD, ejection fraction, echocardiography, pericarditis, coronary artery disease, angioplasty, and angiography). The search and the screening of identified studies for eligibility were conducted by the first author (OB). Our search yielded a total of 2,556 studies (Supplementary File 1). Following deduplication, 2,419 studies underwent further analysis. Cochrane CENTRAL was the priority search source as it is the most comprehensive resource available of RCTs, containing publications from MEDLINE and EMBASE, as well as hand-search results, and gray literature (6, 11). Results of the search were randomly ordered in Excel, by the following method: after exporting the search result as an Excel file from Cochrane CENTRAL, we assigned a random number between 0 and 1 to each record using Excel’s random number generator, then reordered them from the smallest to the highest number (12). As a next step, we screened studies consecutively for eligibility, and the first 250 (∼10%) RCTs matching our pre-specified inclusion criteria were selected (Supplementary File 2). Trials were eligible for inclusion if they were published in the year 2017, were written in English, the described results of an RCT in the field of cardiovascular medicine, and included participants aged ≥ 18 years. Decision on the inclusion of a study was made after a careful consideration of the methodology in the full text.

### Data Extraction

For data extraction, we used a data extraction sheet already tested and described in a previous study (11), data extracting guide available here: https://doi.org/10.1016/j.jpeds.2017.09.014 (12). The following data were extracted: journal type (e.g., specialty cardiovascular, or general medical), the publication details and characteristics of the published trials (such as study design, intervention, trial conduct, study sample, sample size, presence of a data monitoring committee, research outcomes, and conclusions). Further, we collected information about trial registration. Data extraction was completed by two reviewers (OB, OF): the first reviewer extracted the data and then, the second reviewer double-checked the sample. Conflicts were resolved through discussion and by reaching a consensus. Trial registration and protocol availability were investigated by retrieving information from the publications and *via* additional Internet searches (in Google and Google scholar). For the internet searches, we used the trial register number, the investigators’ names, and keywords describing the intervention or the condition.

### Assessment of Methodological Quality and Reporting

We used the Cochrane RoB assessment tool (13) to evaluate the methodological quality of included RCTs. This tool assesses seven domains: (1) random sequence generation (whether the method used to generate the allocation sequence is described in sufficient detail to allow an assessment of whether it should produce comparable groups); (2) allocation concealment (whether the method used to conceal the allocation sequence is described in sufficient detail to determine whether intervention allocations could have been foreseen before or during enrollment); (3) the blinding of participants and personnel; (4) the blinding of outcome assessors; (5) incomplete outcome data (whether attrition and exclusions were reported, whether missing data were balanced across groups or were related to the outcomes); (6) selective reporting (whether pre-specified outcomes were reported in a pre-specified way); and (7) other bias.

We used the Cochrane RoB tool to assess RoB for the primary outcome. When the primary outcome was not clearly defined, we presumed it was the outcome either (1) described under aims/objectives of the study, (2) the outcome used to determine the sample size, or (3) the first outcome reported in the publication (the first applicable was used). One researcher performed a RoB assessment, while a second researcher was assigned to ensure the correctness of the assessments for each study.

Following Cochrane procedures (7), we classified each domain as low, unclear, or high risk. Then, the overall RoB was determined as follows: low when all domains were assessed as low RoB; unclear when at least 1 domain was assessed as unclear and no domains were assessed as high RoB; and high if any domain was assessed as high RoB (7).

### Statistical Analysis

Statistical analyses were conducted by the statistical software R version 4.1.2 (14). The data were analyzed descriptively, using means and standard deviations (SDs) or medians and ranges for continuous variables and proportions for categorical variables. A multivariable logistic regression analysis was conducted to investigate the association between pre-specified study characteristics and the odds of high/unclear RoB. The value of *p* < 0.05 was considered as a significant result.

## Results

### Study Design and Reporting Characteristics of the Study Sample

**FIGURE 1:**
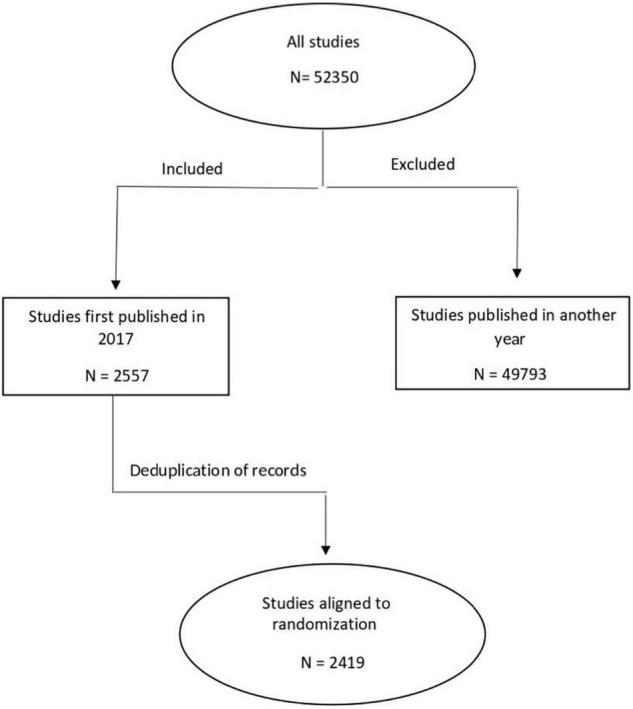
Flowchart of study selection.

Out of the 2,419 studies identified *via* search, we included the first 250 randomly selected trials, which met our search inclusion criteria as shown in Figure 1. The publication and trial characteristics of our sample are shown in Table 1. Most of the included trials had a parallel design (92.4%) and were efficacy trials (94.8%). Overall, 20.8% were placebo-controlled trials. An important part of the results of the trial was published in specialty cardiovascular journals (40.0%). In 139 studies (55.6%), the main goal was to evaluate the effects of pharmacological interventions. All geographic areas were represented; the majority of authors were from Europe (37.4%) and North America (26.0%).

**TABLE 1:** Publication and trial characteristics (*N* = 250).

Study characteristics	*N* (%)
**The geographical location of the corresponding author**	
Asia	65 (26.0%)
North America	69 (27.6%)
Europe (Excluding United Kingdom)	93 (37.2%)
South America	13 (5.2%)
Australia	2 (0.8%)
United Kingdom	8 (3.2%)
**Type of journal**	
Specialty cardiovascular journal	100 (40.0%)
General cardiovascular journal	46 (18.4%)
Specialty medical journal	49 (19.6%)
General medical journal	41 (16.4%)
Non-medical journal	14 (5.6%)
**Study design**	
Parallel	231 (92.4%)
Crossover	15 (6.0%)
Factorial	4 (1.6%)
**Study type**	
Efficacy/Superiority	237 (94.8%)
Equivalence	3 (1.2%)
Non-inferiority	4 (1.6%)
None of the above	6 (2.4%)
**Intervention**	
Drug	139 (55.6%)
Prevention or screening	20 (8.0%)
Device	23 (9.2%)
Other	68 (27.2%)
**Placebo-controlled**	
Yes	68 (27.2%)
No	182 (72.8%)
**Number of centers**	
Multicenter	157 (62.8%)
Single center	93 (37.2%)
**Data Monitoring Committee**	
Yes	105 (42.0%)
No	94 (37.6%)
Unclear	51 (20.4%)
**Funding source**	
Academic or Research institute	94 (37.6%)
Pharmaceutical	48 (19.2%)
Government	24 (9.6%)
Industry for device	10 (4.0%)
No external funding	4 (1.6%)
Private	50 (20.0%)
Unclear	21 (8.4%)
**Primary outcome explicitly specified**	
Yes	157 (62.8%)
No	93 (37.2%)
**Intervention favored**	
Treatment	139 (55.6%)
Control	9 (3.6%)
Non	104 (41.6%)
**Sample size calculation reported**	
Yes	151 (60.4%)
No	99 (39.6%)
**Was there at least one statistically significant outcome?**	
Yes	215 (86.0%)
No	35 (14.0%)
**Was the primary outcome statistically significant?**	
Yes	173 (69.2%)
No	77 (30.8%)
**Overall authors conclusion**	
Positive	170 (68.0%)
Negative	34 (13.6%)
Neutral	46 (18.4%)
**Adverse events**	
Reported	170 (68.0%)
Non-reported	82 (32.8%)
**Trial registered**	
Yes	209
No	41

The funding source was specified in 91.6% of the included trials: most of the trials were funded by an academic grant or a research institute (37.6%), while industrial and pharmaceuticals funding were reported in 23.2% of the trials.

When analyzing the main results of trials, we observed that at least one statistically significant result was reported in 86.0% of the studies; in these studies, the primary outcome was reported to be statistically significant in 69.2% of the cases. The treatment was favored in 55.6% and control in 3.6%. At least one adverse event was reported in 68% of the trials. A data monitoring committee was reported in 42% and sample size calculation in 60.4%. A total of 83.6% of the studies were registered in one of the clinical trials registries out of the 77.5% were registered in clinicaltrials.gov.

### Risk of Bias Assessment

Table 2 shows the RoB assessment results. Overall, 29.2% of the studies were deemed as low RoB, while the remaining studies were at either unclear (39.6%) or high risk (31.2%). We rated the domains sequence generation, allocation concealment, and selective reporting to be the domains most often at high RoB (13.2, 9.6, and 10.4%, respectively).

**TABLE 2:** Risk of bias (RoB) assessments by domain (*N* = 250).

Domain	Risk of bias assessment N (%)		
	High	Unclear	Low
Sequence generation	33(13.2%)	68(27.2%)	149(59.6%)
Allocation concealment	24(9.6%)	51(20.4%)	175(70.0%)
Blinding: participant and personnel	11(4.4%)	112(44.8%)	127(50.8%)
Blinding: outcome assessor	11(4.4%)	33 (13.2%)	206(82.4%)
Incomplete outcome data	8(3.2%)	57(22.8%)	185(74.0%)
Selective reporting	26(10.4%)	67(26.8%)	157(62.8%)
Other bias	36(14.4%)	106(42.4%)	108(42.8%)
Overall RoB	78(31.2%)	99(39.6%)	73(29.2%)

We investigated whether the RoB was associated with the following variables: type of the intervention (drug vs. non-drug); single or multiple study centers; sample size; the presence of a Data Monitoring Committee; statistical significance of the primary outcome and trial registration (Table 3). Of these variables, trial registration influenced overall RoB to the greatest extent (odds ratio [OR] 0.06, 95% CI 0.03–0.31). Drug trials were more likely to have a low RoB than non-drug trials (OR 0.53, 95% CI 0.29–0.97), and multicenter trials more likely than single-center trials (OR 0.39, 95% CI 0.18–0.80). Other investigated variables did not have a significant influence on the RoB.

**TABLE 3:** Multivariable regression analyses for all included trials, and trials with and without stated funding from the industry*.

	All trials (*N* = 250)		Industry-funded trials (*N* = 106)		Non-industry funded trials (*N* = 119)	
	OR (95%CI)	*p*	OR (95%CI)	*p*	OR (95%CI)	*p*
Drug trial (vs. non-drug trial)	0.53 (0.29 – 0.97)	0.04	0.49 (0.18 – 1.27)	0.15	0.50 (0.20 – 1.20)	0.12
Multicentre (vs. single center)	0.39 (0.18 – 0.80)	0.01	0.13 (0.02 – 0.61)	0.02	0.80 (0.32 – 2.00)	0.64
Sample size (>500 vs. smaller)	0.67 (0.34 – 1.31)	0.24	0.60 (0.23 – 1.56)	0.29	1.72 (0.52 – 6.86)	0.40
Data Monitoring Committee (yes vs. no)	0.59 (0.32 – 1.09)	0.09	0.36 (0.13 – 0.96)	0.045	0.91 (0.37 – 2.27)	0.84
Primary outcome statistically significant (vs. not)	0.92 (0.48 – 1.74)	0.80	0.52 (0.81 – 1.11)	0.49	1.36 (0.54 – 3.38)	0.51
Trial registration reported (vs. not reported)	0.06 (0.003 – 0.31)	<0.01	0.19 (0.01 – 1.28)	0.15	1.13 (0.01 – 0.75)	0.06

*Funding was not reported in N = 25 studies.

We observed the following results after investigating individual RoB intems separately: drug trials (OR 0.39, 95% CI 0.22–0.66) and registered trials (OR 0.39, 95% CI 0.18–0.83) were more likely to have low RoB for random sequence generation. Drug trials (OR 0.51, 95% CI 0.28–0.93), registered trials (OR 0.49, 95% CI 0.26–0.91), and multicenter trials (OR 0.49, 95% CI 0.26–0.91) were more likely to have low RoB for allocation concealment, while trials with a statistically significant result were more likely to have unclear or high RoB (OR 2.59, 95% CI 1.34–5.31). Registered trials (OR 0.18, 95% CI 0.06–0.43), trials larger than 500 participants (OR 0.47, 95% CI 0.24–0.92), and trials with a Data Monitoring Committee (OR 0.50, 95% CI 0.28–0.87) had more often low RoB for the blinding of participants and personnel while the blinding of outcome assessors was more often low RoB in multicenter trials (OR 0.42, 95% CI 0.19–0.89) and registered trials (OR 0.27, 95% CI 0.12–0.63). There were no factors that increased the likelihood of low RoB for incomplete outcome data; however, trials with statistically significant results decreased the likelihood of low RoB for incomplete outcome data (OR 2.40, 95% CI 1.18–5.21). Larger trials with more than 500 participants were more likely to have low RoB for selective reporting (OR 0.44, 95% CI 0.19–0.95). Registered trials (OR 0.23, 95% CI 0.08–0.56) and multicenter trials (OR 0.31, 95% CI 0.16–0.59) were more likely to have low RoB for other biases.

### Risk of Bias According to a Funding Source

When funding source was added as an additional independent variable to the multivariable regression model, funding did not seem to influence the likelihood of overall low RoB (industry funding: OR 0.76, 95% CI 0.40–1.45).

In the sub-group of industry-funded trials, multicenter trial and Data Monitoring Committee were factors that increased the likelihood of overall low RoB. None of the investigated factors influenced the overall RoB within the sub-group of trials with non-industry funding (Table 3).

Compared with non-industry funded studies more industry funded studies were rated as low RoB (84.9 vs. 63.9%) and less were rated as unclear (12.3 vs. 26.9%) or high RoB (2.8 vs. 9.2%) for allocation concealment (*p* < 0.001) (Table 4). More industry funded studies were rated low (66.0 vs. 42.9%) and fewer were rated as unclear (33.0 vs. 51.3%) or high risk (0.94 vs. 5.9%) for the blinding of participants and personnel (*p* < 0.001).

**TABLE 4:** Risk of bias assessments by domain in studies funded by the industry or non-industry (*N* = 250).

RoB domain	Funding source (industrial vs. non-industrial)				
	Industrial	*N* (%)	Non- industrial	*N* (%)	*p*-value
Random sequence generation	Low Unclear High	66 (62.3) 32 (30.2) 8 (7.5)	Low Unclear High	71 (59.7) 33 (27.7) 15 (12.6)	0.4533
Allocation concealment	Low Unclear High	90 (84.9) 13 (12.3) 3 (2.8)	Low Unclear High	76 (63.9) 32 (26.9) 11 (9.2)	0.0014*
Blinding: participant and personnel	Low Unclear High	70 (66.0) 35 (33.0) 1 (0.94)	Low Unclear High	51 (42.9) 61 (51.3) 7 (5.9)	0.0001*
Blinding: outcome assessor	Low Unclear High	95 (89.6) 8 (7.5) 3 (2.8)	Low Unclear High	95 (79.8) 19 (16.0) 5 (4.2)	0.1198
Incomplete outcome data	Low Unclear High	86 (81.1) 14 (13.2) 6 (5.7)	Low Unclear High	91 (76.5) 25 (21.0) 3 (2.5)	0.1734
Selective reporting	Low Unclear High	80 (75.5) 23 (21.7) 3 (2.8)	Low Unclear High	86 (72.3) 29 (24.4) 4 (3.4)	0.8598
Other bias	Low Unclear High	55 (51.9) 41 (38.7) 10 (9.4)	Low Unclear High	47 (39.5) 56 (47.1) 16 (13.4)	0.1659
Overall RoB	Low Unclear High	42 (39.6) 41 (38.7) 23 (21.7)	Low Unclear High	30 (25.2) 53 (44.5) 36 (30.3)	0.0587

Statistical analysis was made by regression analysis. *Statistically significant results (p < 0.001).

## Discussion

### Summary of Main Findings

To our knowledge, this is the first research evaluating the RoB and its association with specific trial characteristics in a randomly selected sample of recently published clinical trials in adult cardiovascular disease. Included trials were mainly parallel RCTs investigating the efficacy of an intervention, with a very diverse trial scope and published in a variety of cardiovascular and general medical journals.

Of the 250 studies included, more than 85% have reported at least one statistically significant result, with the primary outcome significant in 69%. Treatment was favored in 55% of the studies, and adverse events were reported in 68%.

Less than one-third of our samples were overall low RoB, while the other two-thirds were unclear or high RoB. Sequence generation, allocation concealment, and selective reporting were the RoB domains most frequently rated at high risk. Trial registration influenced overall RoB to the greatest extent. Drug trials were more likely to be at low RoB than non-drug trials, and multicenter trials were more likely at a low risk RoB than single-center trials.

In the subgroup of industry-funded trials, multicenter trial and Data Monitoring Committee were factors that increased the likelihood of overall low RoB, while none of the investigated factors influenced the overall RoB within the subgroup of non-industry-funded trials. Significant differences were found in the RoB for the domains allocation concealment and the blinding of participants and personnel between industry-funded and non-industry-funded trials, with industry-funded trials more often rated at low risk.

### Strengths and Weaknesses of the Study

We aimed to select a sample of studies representative for all randomized controlled trials published in 2017; we did not exclude published trials based on the country, type of journal, type of participants, or type of intervention. Included trials were randomly selected from all eligible trials. We used the most well-recognized tool for methodological assessment and the results indicated several areas of methodological weaknesses. To increase the reliability of findings, both data extraction and RoB assessment were conducted by two independent researchers.

This study was not pre-registered. Our study is limited by the included trials published in the English language only; conclusions cannot be generalized to trials published in other languages. We could not identify register entries or trial protocols for a subsample of trials, and it was difficult to properly evaluate the selective outcome reporting in these trials. We have not contacted the authors to get additional information about their trials, therefore the assessments are solely based on published information. Additionally, our study focused on the internal validity of trials, but have not assessed factors that may impact the external validity.

### Discussion of Findings Considering Other Studies

Of the 250 analyzed trials, more than two-thirds (70.8%) were at high or unclear RoB, which is consistent with previous study results (15, 16). The RoB domains sequence generation, allocation concealment, and selective reporting were rated most often to be at high risk in our study.

Trial registration had the most beneficial effect on RoB in our study. In previous studies, random sequence generation, allocation concealment, and selective reporting were shown to differ between registered and unregistered gynecology and fertility trials (17). Registration was shown to be a factor influencing all RoB domains except selective outcome reporting in a sample of pediatric research trials (6). Trial registration is a factor that has the potential to facilitate higher methodological quality (18).

We found that drug trials were more likely at a low RoB than non-drug trials. This might be in connection with the strict regulations surrounding drug trials. Differences in protocol quality (19) registration and publication tendencies were described already in previous publications (20, 21), between trials with regulated and non-regulated interventions.

In our study, multi-center trials have demonstrated lower RoB compared with single-center trials. This is consistent with the conclusions of Landoni et al., who underlined bias issues characteristic for single-center trials (e.g., local effect bias, selection and performance bias, detection and reporting bias, analysis and attrition bias, concomitant therapy bias, low fragility index, and publication bias). Caution is advised for the results of single-center RCTs (22).

The source of funding may have an important impact on trial planning, conduct, and reporting (23–25). In our study, we investigated the role of funding sources on RoB by comparing trials both funded or not by the industry. In our study, industry-funded studies were rated more often low for the allocation concealment and the blinding of participants and personnel, as non-industry-funded studies. Our findigs on the higher probability of industry-funded trials to have adequate blinding are supported by the results of a similar study conducted in pediatric RCTs (6, 25). The blinding of participants and personnel may not always be feasible, however, studies should attempt blinding wherever possible.

### Implications for Practice and Future Research

Our results underline the need of further improvement in the process of planning and performing a clinical trial in the field of CVD research. Trial registration was associated with a larger likelihood of low RoB, therefore mandatory trial registration should be endorsed and enforced by ethic committees, funders, and journal editors. Further favorable trial features associated with the lower RoB were multicenter trials, larger trials with more than 500 participants, and trials with a Data monitoring Committee. Trials not funded by the industry were more often at a high RoB for the allocation concealment and the blinding of participants and personnel, indicating, that studies without industry involvement need to pay greater attention to following certain methodological recommendations. The same is applicable for cardiovascular research trials investigating the effects of a non-drug intervention. In the present study, we focused on the methodological quality of CVD research trials but did not assess reporting issues in detail. This may need further assessed in the future.

## Conclusion

Almost two-thirds of RCTs published in 2017 in the field of CVD research were at high or unclear RoB. This indicates a need for more rigorous trial planning and conduct. Prospective trial registration is a factor that predicts the lower RoB.

## Data Availability Statement

The raw data supporting the conclusions of this article will be made available by the authors, without undue reservation.

## Author Contributions

SL conceptualized the research, supervised the research, and reviewed and edited the manuscript. OB and OF performed the research. OB led data extraction and assessment, responsible for data curation, and wrote the first draft of the manuscript. DN conducted statistical analyses. All authors critically revised and approved the final version of the manuscript.

## Conflict of Interest

The authors declare that the research was conducted in the absence of any commercial or financial relationships that could be construed as a potential conflict of interest.

## Publisher’s Note

All claims expressed in this article are solely those of the authors and do not necessarily represent those of their affiliated organizations, or those of the publisher, the editors and the reviewers. Any product that may be evaluated in this article, or claim that may be made by its manufacturer, is not guaranteed or endorsed by the publisher.

## Abbreviations

RCT: randomized-controlled trials
RoB: risk of bias
CVD: cardiovascular disease.
