# Supplementary material for: Methodological Quality and Risk of Bias Assessment of Cardiovascular Disease Research: Analysis of Randomized Controlled Trials Published in 2017
Source: Front Cardiovasc Med. 2022 Mar 17;9:830070. doi: 10.3389/fcvm.2022.830070 (PMC8968023; doi:10.3389/fcvm.2022.830070)
Supplement: Supplementary file 1 [file Data_Sheet_1.PDF]

Search Name: Trials
Date Last saved on: 13/09/2018 08:44:33
Comment: 2017

ID	Search	Hits
#1	(atherosclerosis):ti in Trials	2108
#2	(arrhythmia):ti in Trials	681
#3	(cardiomyopathy):ti in Trials	1522
#4	(heart failure):ti in Trials	15450
#5	(hypertension):ti in Trials	21366
#6	(ischemic heart disease):ti in Trials	980
#7	(heart attack):ti in Trials	303
#8	(angina):ti in Trials	4861
#9	(sudden death):ti in Trials	319
#10	(cardiac arrest):ti in Trials	1459
#11	(hypercholesterolemia):ti in Trials	2352
#12	(High blood pressure):ti in Trials	983
#13	(cardiovascular disease):ti in Trials	3713
#14	(ejection fraction):ti in Trials	1776
#15	(echocardiography):ti in Trials	1151
#16	(angioplasty):ti	3216
#17	#1 or #2 or #3 or #4 or #5 or #6 or #7 or #8 or #9 #10 or #12 or #13 or #14 or #15 with Publication Year from 2017 to 2017, in Trials	2557

Cookies
Our site uses cookies to improve your experience. You can find out more about our use of cookies in About Cookies, including instructions on how to turn off cookies if you wish to do so. By continuing to browse this site you agree to us using cookies as described in About Cookies.

I accept

Cochrane Library

Explore new Cochrane Library features **here**.

We noticed your browser language is English.
You can select your preferred language at the top of any page, and you will see translated Cochrane Review sections in this language. Change to **English.**

Advanced Search

Search manager

Save this search | View saved searches | Search help

Trials

Last saved on: 13/09/2018 08:44:33

2017

View fewer lines | Print

#1	(atherosclerosis):ti in Trials	S MeSH	Limits	2108
#2	(arrhythmia):ti in Trials		Limits	681
#3	(cardiomyopathy):ti in Trials		Limits	1522
#4	(heart failure):ti in Trials		Limits	15450
#5	(hypertension):ti in Trials		Limits	21366

#6	(ischemic heart disease):ti	in Trials	Limits	980
#7	(heart attack):ti	in Trials	Limits	303
#8	(angina):ti	in Trials	Limits	4861
#9	(sudden death):ti	in Trials	Limits	319
#10	(cardiac arrest):ti	in Trials	Limits	1459
#11	(hypercholesterolemia):ti	in Trials	Limits	2352
#12	(High blood pressure):ti	in Trials	Limits	983
#13	(cardiovascular disease):ti	in Trials	Limits	3713
#14	(ejection fraction):ti	in Trials	Limits	1776
#15	(echocardiography):ti	in Trials	Limits	1151
#16	(angioplasty):ti		Limits	3216
#17	#1 or #2 or #3 or #4 or #5 or #6 or #7 or #8 or #9 #10 or #12 or #13 or [illegible]	with Publication Year from 2017 to 2017, in Trials	Limits	2557
#18	Type a search term or use the S or MeSH [illegible]	S MeSH	Limits	N/A

Clear all

Highlight orphan lines

Save this search | View saved searches | ? Search help

Trials

Last saved on: 13/09/2018 08:44:33

2017

View fewer lines | Print
