# Supplementary material for: Methodological Quality and Risk of Bias Assessment of Cardiovascular Disease Research: Analysis of Randomized Controlled Trials Published in 2017
Source: Front Cardiovasc Med. 2022 Mar 17;9:830070. doi: 10.3389/fcvm.2022.830070 (PMC8968023; doi:10.3389/fcvm.2022.830070)
Supplement: Supplementary file 2 [file Data_Sheet_2.PDF]

Number	Authors	Title	Journal
1.	Magnuson EA, Li H, Wang K, Vilain K, Shafiq A, Bonaca MP, Bhatt DL, Cohen M, Steg PG, Storey RF, Braunwald E, Sabatine MS, Cohen DJ; PEGASUS-TIMI 54 Trial Investigators.	Cost-Effectiveness of Long-Term Ticagrelor in Patients With Prior Myocardial Infarction Results From the PEGASUS-TIMI 54 Trial	J Am Coll Cardiol. 2017 Aug 1;70(5):527-538. doi: 10.1016/j.jacc.2017.05.063. PMID: 28750695.
2.	St Pierre M, Luetcke B, Strembski D, Schmitt C, Breuer G.	The effect of an electronic cognitive aid on the management of ST-elevation myocardial infarction during caesarean section: a prospective randomised	*BMC Anesthesiol*. 2017;17(1):46. Published 2017 Mar 20. doi:10.1186/s12871-017-0340-4
3.	Meischke H, Painter IS, Stangenes SR, Weaver MR, Fahrenbruch CE, Rea T, Turner AM.	Simulation training to improve 9-1-1 dispatcher identification of cardiac arrest: A randomized controlled trial.	Resuscitation. 2017 Oct;119:21-26. doi:10.1016/j.resuscitation.2017.07.025. Epub 2017 Jul 29. PMID: 28760696.
4.	Priti K, Ranwa BL, Gokhroo RK, Kishore K, Bisht DS, Gupta S.	Ivabradine vs metoprolol in patients with acute inferior wall myocardial infarction- "Expanding arena for ivabradine	Cardiovasc Ther. 2017 Aug;35(4). doi: 10.1111/1755-5922.12266. PMID: 28423233. India, English
5.	Kuramitsu, S	Effect of sitagliptin on plaque changes in coronary artery following acute coronary syndrome in diabetic patients: the ESPECIAL-ACS study	Journal of Cardiology , Volume69, Issue 1, Pages 369-376, Japan , English
6.	Jayo-Montoya, Ja: Aispuru, Gr: Villar-Zabala, B: Matajira-Chia, T: Gallardo-Lobo, R:	Effects of different high intensity aerobic interval training programs with Mediterranean diet recommendations in post-myocardial infarct patients: preliminary results of INTERFARCT controlled trial s	European journal of preventive cardiology

	Maldonado-Martin, S		
7.	Pan Y, Lu Z, Hang J, Ma S, Ma J, Wei M.	Effects of Low-Dose Recombinant Human Brain Natriuretic Peptide on Anterior Myocardial Infarction Complicated by Cardiogenic Shock.	Braz J Cardiovasc Surg. 2017 Mar-Apr;32(2):96-103. doi: 10.21470/1678-9741-2016-0007. PMID: 28492790; PMCID: PMC5409251
8.	Kopec M, Duma A, Helwani MA, Brown J, Brown F, Gage BF, Gibson DW, Miller JP, Novak E, Jaffe AS, Apple FS, Scott MG, Nagele P.	Improving Prediction of Postoperative Myocardial Infarction With High-Sensitivity Cardiac Troponin T and NT-proBNP	Anesth Analg. 2017 Feb;124(2):398-405. doi: 10.1213/ANE.0000000000001736. PMID: 28002165; PMCID: PMC5243152.
9.	Scales DC, Cheskes S, Verbeek PR, Pinto R, Austin D, Brooks SC, Dainty KN, Goncharenko K, Mamdani M, Thorpe KE, Morrison LJ;	Prehospital cooling to improve successful targeted temperature management after cardiac arrest: A randomized controlled trial	Resuscitation. 2017 Dec;121:187-194. doi: 10.1016/j.resuscitation.2017.10.002. Epub 2017 Oct 5. PMID: 28988962.
10.	OJHA RAKESH	A RANDOMIZED ACTIVE CONTROLLED CLINICAL STUDY TO EVALUATE EFFICACY AND SAFETY OF RESVERATROL AS AN ADJUVANT THERAPY IN PATIENTS WITH HYPERTENSION	Asian journal of Pharmaceutical and Clinical research, 2017;10;1, INDIA, English
11.	Omar HR, Charnigo R, Guglin M.	Ratio of Systolic Blood Pressure to Right Atrial Pressure, a Novel Marker to Predict Morbidity and Mortality in Acute Systolic Heart Failure.	Am J Cardiol. 2017 Apr 1;119(7):1061-1068. doi: 10.1016/j.amjcard.2016.11.062. Epub 2017 Jan 6. PMID: 28215413.
12.	Mazereeuw G, Herrmann N,	Oxidative stress predicts depressive symptom changes with omega-3 fatty acid treatment in coronary artery disease patients.	Brain Behav Immun. 2017 Feb;60:136-141. doi:

	Andreazza AC, Scola G, Ma DWL, Oh PI, Lanctôt KL.		10.1016/j.bbi.2016.10.005. Epub 2016 Oct 11. PMID: 27742581.
13.	Han X, Zhang Y, Dong L, Fang L, Chai Y, Niu M, Yu Y, Liu L, Yang X, Qu S, Li S.	Treatment of Pulmonary Arterial Hypertension Using Initial Combination Therapy of Bosentan and Iloprost,	Respir Care. 2017 Apr;62(4):489-496. doi: 10.4187/respcare.05280. Epub 2017 Jan 24. PMID: 28119496.
14.	Kim NH, D'Armini AM, Grimminger F, Grünig E, Hoeper MM, Jansa P, Mayer E, Neurohr C, Simonneau G, Torbicki A, Wang C, Fritsch A, Davie N, Ghofrani HA.	Haemodynamic effects of riociguat in inoperable/recurrent chronic thromboembolic pulmonary hypertension. Kim NH, D'Armini AM, Grimminger F, Grünig E, Hoeper MM, Jansa P, Mayer E, Neurohr C, Simonneau G, Torbicki A, Wang C, Fritsch A, Davie N, Ghofrani HA	Heart. 2017 Apr;103(8):599-606. doi: 10.1136/heartjnl-2016-309621. Epub 2016 Dec 23. PMID: 28011757; PMCID: PMC5529957. Multicenter,
15.	Taborsky M, Ostadal P, Adam T, Moravec O, Gloger V, Schee A, Skala T. Bratisl Lek Listy.	Red or white wine consumption effect on atherosclerosis in healthy individuals (In Vino Veritas study).	2017;118(5):292-298. doi: 10.4149/BLL_2017_072. PMID: 28516793. Czech
16.	Tani S, Asayama K, Oiwa K, Harasawa S, Okubo K, Takahashi A, Tanabe A, Ohkubo T, Hirayama A, Kushiro T.	The effects of increasing calcium channel blocker dose vs. adding a diuretic to treatment regimens for patients with uncontrolled hypertension.	Hypertens Res. 2017 Oct 5;40(10):892-898. doi: 10.1038/hr.2017.56. Epub 2017 Apr 27. PMID: 28446804.
17.	Wang J, Ai XB, Wang F, Zou YW, Li L, Yi XL.	Efficacy of ezetimibe combined with atorvastatin in the treatment of carotid artery plaque in patients with type 2 diabetes mellitus complicated with coronary heart disease.	Int Angiol. 2017 Oct;36(5):467-473. doi: 10.23736/S0392-9590.17.03818-4.

			Epub 2017 Jun 21. PMID: 28641407.
18.	Rudolph TK, Fuchs A, Klinke A, Schlichting A, Friedrichs K, Hellmich M, Mollenhauer M, Schwedhelm E, Baldus S, Rudolph V.	Prasugrel as opposed to clopidogrel improves endothelial nitric oxide bioavailability and reduces platelet-leukocyte interaction in patients with unstable angina pectoris: A randomized controlled trial.	Int J Cardiol. 2017 Dec 1;248:7-13. doi: 10.1016/j.ijcard.2017.06.099. Epub 2017 Jul 1. PMID: 28709700.
19.	Auscher S, Løgstrup BB, Møller JE, Vinther KH, Lambrechtsen J, Egstrup K.	Effects of Intensive Statin Therapy on Left Ventricular Function in Patients with Myocardial Infarction and Abnormal Glucose Tolerance.	Cardiology. 2017;138(1):16-25. doi: 10.1159/000469657. Epub 2017 May 18. PMID: 28514784.
20.	Lybeck A, Friberg H, Aneman A, Hassager C, Horn J, Kjærgaard J, Kuiper M, Nielsen N, Ullén S, Wise MP, Westhall E, Cronberg T; TTM-trial Investigators	Prognostic significance of clinical seizures after cardiac arrest and target temperature management	Resuscitation. 2017 May;114:146-151. doi: 10.1016/j.resuscitation.2017.01.017. Epub 2017 Feb 3. PMID: 28163232.
21.	Gallagher BD, Moise N, Haerizadeh M, Ye S, Medina V, Kronish IM.	Telemonitoring Adherence to Medications in Heart Failure Patients (TEAM-HF): A Pilot Randomized Clinical Trial	Clinical Trial. J Card Fail. 2017 Apr;23(4):345-349. doi: 10.1016/j.cardfail.2016.11.001. Epub 2016 Nov 3. PMID: 27818309; PMCID: PMC5380538.
22.	Jaguszewski M, Aloysius R, Wang W, Bezerra HG, Hill J, De Winter RJ, Karjalainen	The REMEDEE-OCT Study: An Evaluation of the Bioengineered COMBO Dual-Therapy CD34 Antibody-Covered Sirolimus-Eluting Coronary Stent Compared With a Cobalt-Chromium Everolimus-Eluting Stent in Patients With Acute Coronary Syndromes: Insights From	JACC Cardiovasc Interv. 2017 Mar 13;10(5):489-499. doi: 10.1016/j.jcin.2016.11.040. PMID: 28279316.

	PP, Verheye S, Wijns W, Lüscher TF, Joner M, Costa M, Landmesser U.	Optical Coherence Tomography Imaging Analysis.	
23.	Gotsman I, Ezra O, Hirsh Raccah B, Admon D, Lotan C, Dekeyser Ganz F.	Patient-Specific Tailored Intervention Improves INR Time in Therapeutic Range and INR Variability in Heart Failure Patients.	Am J Med. 2017 Aug;130(8):982-989. doi: 10.1016/j.amjmed.2017.02.030. Epub 2017 Mar 24. PMID: 28344144.
24.	Packer M, McMurray JJV, Krum H, Kiowski W, Massie BM, Caspi A, Pratt CM, Petrie MC, DeMets D, Kobrin I, Roux S, Swedberg K; ENABLE Investigators and Committees.	Long-Term Effect of Endothelin Receptor Antagonism With Bosentan on the Morbidity and Mortality of Patients With Severe Chronic Heart Failure: Primary Results of the ENABLE Trials.	JACC Heart Fail. 2017 May;5(5):317-326. doi: 10.1016/j.jchf.2017.02.021. PMID: 28449795.
25.	Boriani G, Da Costa A, Quesada A, Ricci RP, Favale S, Boscolo G, Clementy N, Amori V, Mangoni di S Stefano L, Burri H;	MORE-CARE Study Investigators. Effects of remote monitoring on clinical outcomes and use of healthcare resources in heart failure patients with biventricular defibrillators: results of the MORE-CARE multicentre randomized controlled trial.	Eur J Heart Fail. 2017 Mar;19(3):416-425. doi: 10.1002/ejhf.626. Epub 2016 Aug 28. PMID: 27568392.
26.	Packer M, Pitt B, Rouleau JL, Swedberg K, DeMets DL,	Fisher L. Long-Term Effects of Flosequinan on the Morbidity and Mortality of Patients With Severe Chronic Heart Failure: Primary Results of the PROFILE Trial After 24 Years. JACC Heart Fail.	2017 Jun;5(6):399-407. doi: 10.1016/j.jchf.2017.03.003. Epub 2017 May 10. PMID: 28501522.
27.	Kuck KH, Tilz RR, Deneke T, Hoffmann BA,	Impact of Substrate Modification by Catheter Ablation on Implantable Cardioverter-Defibrillator Interventions in Patients With Unstable Ventricular Arrhythmias and Coronary Artery Disease:	Circ Arrhythm Electrophysiol. 2017 Mar;10(3):e004422. doi:

	Ventura R, Hansen PS, Zarse M, Hohnloser SH, Kautzner J, Willems S; SMS Investigators.	Results From the Multicenter Randomized Controlled SMS (Substrate Modification Study).	10.1161/CIRCEP.116.004422. PMID: 28292751.
28.	Dufour R, Bergeron J, Gaudet D, Weiss R, Hovingh GK, Qing Z, Yang F, Andisik M, Torri A, Pordy R, Gipe DA.	Open-label therapy with alirocumab in patients with heterozygous familial hypercholesterolemia: Results from three years of treatment.	Int J Cardiol. 2017 Feb 1;228:754-760. doi: 10.1016/j.ijcard.2016.11.046. Epub 2016 Nov 9. PMID: 27886619.
29.	Kim NH, D'Armini AM, Grimminger F, Grünig E, Hoeper MM, Jansa P, Mayer E, Neurohr C, Simonneau G, Torbicki A, Wang C, Fritsch A, Davie N, Ghofrani HA.	Haemodynamic effects of riociguat in inoperable/recurrent chronic thromboembolic pulmonary hypertension.	Heart. 2017 Apr;103(8):599-606. doi: 10.1136/heartjnl-2016-309621. Epub 2016 Dec 23. PMID: 28011757; PMCID: PMC5529957.
30.	Damman K, Valente MAE, van Veldhuisen DJ, Cleland JGF, O'Connor CM, Metra M, Ponikowski P, Cotter G, Davison B, Givertz MM, Bloomfield DM, Hillege HL, Voors AA.	Plasma Neutrophil Gelatinase-Associated Lipocalin and Predicting Clinically Relevant Worsening Renal Function in Acute Heart Failure.	Int J Mol Sci. 2017 Jul 8;18(7):1470. doi: 10.3390/ijms18071470. PMID: 28698481; PMCID: PMC5535961.

31.	Wang X, Hu D, Dang S, Huang H, Huang CX, Yuan MJ, Tang YH, Zheng QS, Yin F, Zhang S, Zhang BL, Gao RL;	Efficacy and Safety of Shensong Yangxin Capsules for Frequent VPCs in Congestive Heart Failure Study Group. Effects of Traditional Chinese Medicine Shensong Yangxin Capsules on Heart Rhythm and Function in Congestive Heart Failure Patients with Frequent Ventricular Premature Complexes: A Randomized, Double-blind, Multicenter Clinical Trial.	Chin Med J (Engl). 2017 Jul 20;130(14):1639-1647. doi: 10.4103/0366-6999.209906. PMID: 28685712; PMCID: PMC5520549.
32.	Grübler MR, Gaksch M, Kienreich K, Verheyen ND, Schmid J, Müllner C, Richtig G, Scharnagl H, Trummer C, Schwetz V, Meinitzer A, Pieske B, März W, Tomaschitz A, Pilz S.	Effects of Vitamin D3 on asymmetric- and symmetric dimethylarginine in arterial hypertension.	J Steroid Biochem Mol Biol. 2018 Jan;175:157-163. doi: 10.1016/j.jsbmb.2016.12.014. Epub 2016 Dec 24. PMID: 28027911.
33.	Shi R, Liu K, Shi D, Liu Q, Chen X.	Effects of Amlodipine and Valsartan on Blood Pressure Variability and Pulse Wave Velocity in Hypertensive Patients.	Am J Med Sci. 2017 Jan;353(1):6-11. doi: 10.1016/j.amjms.2016.10.005. Epub 2016 Oct 27. PMID: 28104105.
34.	de Leeuw PW, Fagard R, Kroon AA.	The effects of missed doses of amlodipine and losartan on blood pressure in older hypertensive patients.	Hypertens Res. 2017 Jun;40(6):568-572. doi: 10.1038/hr.2016.190. Epub 2017 Jan 19. PMID: 28100922.
35.	. Steinhoff G, Nesteruk J, Wolfien M, Kundt G; EBioMedicine.	PERFECT Trial Investigators Group, Börgermann J, David R, Garbade J, Große J, Haverich A, Hennig H, Kaminski A, Lotz J, Mohr FW, Müller P, Oostendorp R, Ruch U, Sarikouch S, Skorska A, Stamm C, Tiedemann G, Wagner FM, Wolkenhauer O. Cardiac Function Improvement and Bone Marrow Response -: Outcome Analysis of the Randomized	017 Aug;22:208-224. doi: 10.1016/j.ebiom.2017.07.022. Epub 2017 Jul 29. PMID: 28781130; PMCID: PMC5552265.Germany, English

		PERFECT Phase III Clinical Trial of Intramyocardial CD133+ Application After Myocardial Infarction2	
36.	Massberg S, Byrne RA, Kastrati A, Schulz S, Pache J, Hausleiter J, Ibrahim T, Fusaro M, Ott I, Schömig A, Laugwitz KL, Mehilli J;	Intracoronary Stenting and Angiographic Results: Test Efficacy of Sirolimus- and Probucol-Eluting Versus Zotarolimus-Eluting Stents (ISAR-TEST 5) Investigators. Polymer-free sirolimus- and probucol-eluting versus new generation zotarolimus-eluting stents in coronary artery disease: the Intracoronary Stenting and Angiographic Results: Test Efficacy of Sirolimus- and Probucol-Eluting versus Zotarolimus-eluting Stents (ISAR-TEST 5) trial..	Circulation. 2011 Aug 2;124(5):624-32. doi: 10.1161/CIRCULATIONAHA.111.026732. Epub 2011 Jul 18. PMID: 21768546.
37.	Fujiwara T, Tomitani N, Kanegae H, Kario K.	Comparative effects of valsartan plus either cilnidipine or hydrochlorothiazide on home morning blood pressure surge evaluated by information and communication technology-based nocturnal home blood pressure monitoring.	J Clin Hypertens (Greenwich). 2018 Jan;20(1):159-167. doi: 10.1111/jch.13154. Epub 2018 Jan 5. PMID: 29316213; PMCID: PMC8030821.
38.	Derkach A, Sampson J, Joseph J, Playdon MC, Stolzenberg-Solomon RZ.	Effects of dietary sodium on metabolites: the Dietary Approaches to Stop Hypertension (DASH)-Sodium Feeding Study.	Am J Clin Nutr. 2017 Oct;106(4):1131-1141. doi: 10.3945/ajcn.116.150136. Epub 2017 Aug 30. PMID: 28855223; PMCID: PMC5611778.
39.	Safdar B, D'Onofrio G, Dziura J, Russell RR, Johnson C, Sinusas AJ.	Ranolazine and Microvascular Angina by PET in the Emergency Department: Results From a Pilot Randomized Controlled Trial.	Clin Ther. 2017 Jan;39(1):55-63. doi: 10.1016/j.clinthera.2016.12.002. Epub 2017 Jan 9. PMID: 28081848.
40.	Toyoda S, Haruyama A, Inami S, Amano H, Arikawa T, Sakuma M, Abe S, Tanaka A, Node K, Inoue T.	Protective effects of bisoprolol against myocardial injury and pulmonary dysfunction in patients with chronic heart failure.	Int J Cardiol. 2017 Jan 1;226:71-76. doi: 10.1016/j.ijcard.2016.10.046. Epub 2016 Oct 22. PMID: 27792991.
41.	Sanati HR, Zahedmehr A, Firouzi A, Farrashi	Intracoronary versus Intravenous eptifibatide during percutaneous coronary intervention for acute ST-segment	Cardiovasc Interv Ther. 2017 Oct;32(4):351-357. doi:

	M, Amin K, Peighambari MM, Shakerian F, Kiani R.	elevation myocardial infarction; a randomized controlled trial.	10.1007/s12928-016-0418-9. Epub 2016 Aug 17. PMID: 27534402.
42.	Glantz H, Johansson MC, Thunström E, Guron CW, Uzel H, Saygin M, Herlitz J, Peker Y.	Effect of CPAP on diastolic function in coronary artery disease patients with nonsleepy obstructive sleep apnea: A randomized controlled trial.	Int J Cardiol. 2017 Aug 15;241:12-18. doi: 10.1016/j.ijcard.2017.03.100. Epub 2017 Mar 25. PMID: 28408103.
43.	Krustrup P, Skoradal MB, Randers MB, Weihe P, Uth J, Mortensen J, Mohr M.	Broad-spectrum health improvements with one year of soccer training in inactive mildly hypertensive middle-aged women.	Scand J Med Sci Sports. 2017 Dec;27(12):1893-1901. doi: 10.1111/sms.12829. Epub 2017 Jan 25. PMID: 28124381.
44.	Cheung AK, Rahman M, Reboussin DM, Craven TE, Greene T, Kimmel PL, Cushman WC, Hawfield AT, Johnson KC, Lewis CE, Oparil S, Rocco MV, Sink KM, Whelton PK, Wright JT Jr, Basile J, Beddhu S, Bhatt U, Chang TI, Chertow GM, Chonchol M, Freedman BI, Haley W, Ix JH, Katz LA, Killeen AA, Papademetriou V,	Effects of Intensive BP Control in CKD.	J Am Soc Nephrol. 2017 Sep;28(9):2812-2823. doi: 10.1681/ASN.2017020148. Epub 2017 Jun 22. PMID: 28642330; PMCID: PMC5576945. USA, English

	Ricardo AC, Servilla K, Wall B, Wolfgram D, Yee J; SPRINT Research Group.		
45.	Mehra MR, Naka Y, Uriel N, Goldstein DJ, Cleveland JC Jr, Colombo PC, Walsh MN, Milano CA, Patel CB, Jorde UP, Pagani FD, Aaronson KD, Dean DA, McCants K, Itoh A, Ewald GA, Horstmanshof D, Long JW, Salerno C; MOMENTUM 3 Investigators.	A Fully Magnetically Levitated Circulatory Pump for Advanced Heart Failure.	N Engl J Med. 2017 Feb 2;376(5):440-450. doi: 10.1056/NEJMoa1610426. Epub 2016 Nov 16. PMID: 27959709.
46.	Lee JH, Kim SH, Choi DJ, Tahk SJ, Yoon JH, Choi SW, Hong TJ, Kim HS.	Efficacy and tolerability of two different formulations of atorvastatin in Korean patients with hypercholesterolemia: a multicenter, prospective, randomized clinical trial..	Drug Des Devel Ther. 2017 Aug 2;11:2277-2285. doi: 10.2147/DDDT.S112241. PMID: 28814835; PMCID: PMC5546732.Korea, English
47.	Xiao S, Zhang M, Liang Y, Wang D.	Celastrol synergizes with oral nifedipine to attenuate hypertension in preeclampsia: a randomized, placebo-controlled, and double blinded trial.	J Am Soc Hypertens. 2017 Sep;11(9):598-603. doi: 10.1016/j.jash.2017.07.004. Epub 2017 Jul 20. PMID: 28757108.
48.	Campo G, Vieceli	Biological effects of ticagrelor over clopidogrel in patients with stable coronary	Thromb Haemost. 2017 Mar

	Dalla Sega F, Pavasini R, Aquila G, Gallo F, Fortini F, Tonet E, Cimaglia P, Del Franco A, Pestelli G, Pecoraro A, Contoli M, Balla C, Biscaglia S, Rizzo P, Ferrari R.	artery disease and chronic obstructive pulmonary disease.	23;117(6):1208-1216. doi: 10.1160/TH16-12-0973. Epub 2017 Mar 23. PMID: 28331925; PMCID: PMC6291849.
49.	Choudhury T, Mozid A, Hamshere S, Yeo C, Pellaton C, Arnous S, Saunders N, Brookman P, Jain A, Locca D, Archbold A, Knight C, Wragg A, Davies C, Mills P, Parmar M, Rothman M, Choudry F, Jones DA, Agrawal S, Martin J, Mathur A.	An exploratory randomized control study of combination cytokine and adult autologous bone marrow progenitor cell administration in patients with ischaemic cardiomyopathy: the REGENERATE-IHD clinical trial.	Eur J Heart Fail. 2017 Jan;19(1):138-147. doi: 10.1002/ejhf.676. Epub 2016 Oct 28. PMID: 27790824; PMCID: PMC5248636.
50.	Matos-Garcia BC, Rocco IS, Maiorano LD, Peixoto TCA, Moreira RSL, Carvalho ACC, Catai AM, Arena R,	Home-Based Walking Program Improves Respiratory Endurance in Patients With Acute Myocardial Infarction: A Randomized Controlled	Trial. Can J Cardiol. 2017 Jun;33(6):785-791. doi: 10.1016/j.cjca.2016.12.004. Epub 2016 Dec 10. PMID: 28545625.
51.	Matsue Y, Yoshioka K, Suzuki M, Torii S, Yamaguchi S, Fukamizu S, Ono Y,	Prognostic importance of sodium level trajectory in acute heart failure.	Heart Vessels. 2017 Dec;32(12):1498-1505. doi: 10.1007/s00380-017-1020-5. Epub 2017 Jul 11. PMID: 28698994.

	Fujii H, Kitai T, Nishioka T, Sugi K, Onishi Y, Noda M, Kagiyama N, Satoh Y, Yoshida K, Goldsmith SR.		
52.	Steinhart BD, Levy P, Vandenberghe H, Moe G, Yan AT, Cohen A, Thorpe KE, McGowan M, Mazer CD.	A Randomized Control Trial Using a Validated Prediction Model for Diagnosing Acute Heart Failure in Undifferentiated Dyspneic Emergency Department Patients-Results of the GASP4Ar Study.	J Card Fail. 2017 Feb;23(2):145-152. doi: 10.1016/j.cardfail.2016.08.007. Epub 2016 Aug 24. PMID: 27565045. Canada/USA, English
53.	Li Y, Yang Y, Li Q, Yang X, Wang Y, Ku WL, Li H.	The impact of the improvement of insomnia on blood pressure in hypertensive patients.	J Sleep Res. 2017 Feb;26(1):105-114. doi: 10.1111/jsr.12411. Epub 2016 Apr 20. PMID: 27095167.
54.	Zamani P, Tan V, Soto-Calderon H, Beraun M, Brandimarto JA, Trieu L, Varakantam S, Doulias PT, Townsend RR, Chittams J, Margulies KB, Cappola TP, Poole DC, Ischiropoulos H, Chirinos JA.	Pharmacokinetics and Pharmacodynamics of Inorganic Nitrate in Heart Failure With Preserved Ejection Fraction.	Circ Res. 2017 Mar 31;120(7):1151-1161. doi: 10.1161/CIRCRESAHA.116.309832. Epub 2016 Dec 7. PMID: 27927683; PMCID: PMC5376233.
55.	Valborgland T, Isaksen K, Munk PS, Grabowski ZP, Larsen AI.	Impact of an exercise training program on cardiac neuronal function in heart failure patients on optimal medical therapy : A randomized Iodine-123 metaiodobenzylguanidine scintigraphy study.	J Nucl Cardiol. 2018 Aug;25(4):1164-1171. doi: 10.1007/s12350-016-0724-8. Epub 2017 Jan 17. PMID: 28097476.

56.	Kalter-Leibovici O, Freimark D, Freedman LS, Kaufman G, Ziv A, Murad H, Benderly M, Silverman BG, Friedman N, Cukierman-Yaffe T, Asher E, Grupper A, Goldman D, Amitai M, Matetzky S, Shani M, Silber H;	Israel Heart Failure Disease Management Study (IHF-DMS) investigators. Disease management in the treatment of patients with chronic heart failure who have universal access to health care: a randomized controlled trial.	BMC Med. 2017 May 1;15(1):90. doi: 10.1186/s12916-017-0855-z. PMID: 28457231; PMCID: PMC5410698.
57.	Derosa G, Maffioli P, D'Avino M, Sala C, Mugellini A, Vulpis V, Felis S, Guasti L, Sarzani R, Bestetti A, Vanasia M, Gaudio G;	ESCAPE-IT Trial Investigators group. Efficacy and safety of two dosages of canrenone as add-on therapy in hypertensive patients taking ace-inhibitors or angiotensin II receptor blockers and hydrochlorothiazide at maximum dosage in a randomized clinical trial: The ESCAPE-IT trial.	Cardiovasc Ther. 2017 Feb;35(1):47-54. doi: 10.1111/1755-5922.12235. PMID: 27860389; PMCID: PMC6680336.
58.	Peters CD, Mathiassen ON, Vase H, Bech Nørgaard J, Christensen KL, Schroeder AP, Rickers HJVH, Opstrup UK, Poulsen PL, Langfeldt S, Andersen G, Hansen KW, Bøtker HE, Engholm M, Bertelsen JB, Pedersen	The effect of renal denervation on arterial stiffness, central blood pressure and heart rate variability in treatment resistant essential hypertension: a substudy of a randomized sham-controlled double-blinded trial (the ReSET trial).	Blood Press. 2017 Dec;26(6):366-380. doi: 10.1080/08037051.2017.1368368. Epub 2017 Aug 23. PMID: 28830251.

	EB, Kaltoft A, Buus NH		
59.	Moraes IG, Kimoto KM, Fernandes MB, Grams ST, Yamaguti WP.	Adjunctive Use of Noninvasive Ventilation During Exercise in Patients With Decompensated Heart Failure.	Am J Cardiol. 2017 Feb 1;119(3):423-427. doi: 10.1016/j.amjcard.2016.10.025. Epub 2016 Nov 1. PMID: 27939229.
60.	Patel RB, Tannenbaum S, Viana-Tejedor A, Guo J, Im K, Morrow DA, Scirica BM	Serum potassium levels, cardiac arrhythmias, and mortality following non-ST-elevation myocardial infarction or unstable angina: insights from MERLIN-TIMI 36	. Eur Heart J Acute Cardiovasc Care. 2017 Feb;6(1):18-25. doi: 10.1177/2048872615624241. Epub 2016 Sep 20. PMID: 26714972; PMCID: PMC5410890.
61.	Giannitsis E, Wallentin L, James SK, Bertilsson M, Siegbahn A, Storey RF, Husted S, Cannon CP, Armstrong PW, Steg PG, Katus HA;	PLATO investigators. Outcomes after planned invasive or conservative treatment strategy in patients with non-ST-elevation acute coronary syndrome and a normal value of high sensitivity troponin at randomisation: A Platelet Inhibition and Patient Outcomes (PLATO) trial biomarker substudy.	Eur Heart J Acute Cardiovasc Care. 2017 Sep;6(6):500-510. doi: 10.1177/2048872616641901. Epub 2016 Apr 4. PMID: 27044282.
62.	Vizza CD, Sastry BK, Safdar Z, Harnisch L, Gao X, Zhang M, Lamba M, Jing ZC.	Efficacy of 1, 5, and 20 mg oral sildenafil in the treatment of adults with pulmonary arterial hypertension: a randomized, double-blind study with open-label extension.	BMC Pulm Med. 2017 Feb 23;17(1):44. doi: 10.1186/s12890-017-0374-x. PMID: 28228114; PMCID: PMC5322647.
63.	Consuegra-Sanchez L, Piccolo R, Gonzalez-Gonzalez J, Garcia-Camarero T, Del Mar Garcia-Saiz M, Aldea-Perona A, Reiter RJ; MARIA	Dominguez-Rodriguez A, Abreu-Gonzalez P, de la Torre-Hernandez JM, Usefulness of Early Treatment With Melatonin to Reduce Infarct Size in Patients With ST-Segment Elevation Myocardial Infarction Receiving Percutaneous Coronary Intervention (From the Melatonin Adjunct in the Acute Myocardial Infarction Treated With Angioplasty Trial).	Am J Cardiol. 2017 Aug 15;120(4):522-526. doi: 10.1016/j.amjcard.2017.05.018. Epub 2017 May 30. PMID: 28645475.

	Investigators.		
64.	Qian G, Yang YQ, Dong W, Cao F, Chen YD.	Comparison of Iodixanol and Iopromide in Patients With Renal Insufficiency and Congestive Heart Failure Undergoing Coronary Angiography by Hemodynamic Monitoring.	Angiology. 2017 Nov;68(10):907-913. doi: 10.1177/0003319717701868. Epub 2017 Apr 12. PMID: 28401790.
65.	Azevedo ER, Mak S, Floras JS, Parker JD.	Acute effects of angiotensin-converting enzyme inhibition versus angiotensin II receptor blockade on cardiac sympathetic activity in patients with heart failure.	Am J Physiol Regul Integr Comp Physiol. 2017 Oct 1;313(4):R410-R417. doi: 10.1152/ajpregu.00095.2017. Epub 2017 Jul 5. PMID: 28679681.
66.	Bertelsen JB, Refsgaard J, Kanstrup H, Johnsen SP, Qvist I, Christensen B, Christensen KL.	Cardiac rehabilitation after acute coronary syndrome comparing adherence and risk factor modification in a community-based shared care model versus hospital-based care in a randomised controlled trial with 12 months of follow-up.	Eur J Cardiovasc Nurs. 2017 Apr;16(4):334-343. doi: 10.1177/1474515116666781. Epub 2016 Sep 23. PMID: 27566597.
67.	Gotsman I, Ezra O, Hirsh Raccah B, Admon D, Lotan C, Dekeyser Ganz F.	Patient-Specific Tailored Intervention Improves INR Time in Therapeutic Range and INR Variability in Heart Failure Patients.	Am J Med. 2017 Aug;130(8):982-989. doi: 10.1016/j.amjmed.2017.02.030. Epub 2017 Mar 24. PMID: 28344144.
68.	Hua K, Hao G, Li W.	Cardiovascular outcomes of lifestyle intervention in hypertensive patients with antihypertensive agents.	Int J Cardiol. 2017 Jan 15;227:751-756. doi: 10.1016/j.ijcard.2016.10.062. Epub 2016 Oct 27. PMID: 27810294.
69.	Noronha Neto C, C., Maia, S. S., Katz, L., Coutinho, I. C., Souza, A. R., & Amorim, M. M. (2017).	Clonidine versus Captopril for Severe Postpartum Hypertension: A Randomized Controlled Trial.	*PloS one*, *12*(1), e0168124. https://doi.org/10.1371/journal.pone.0168124

70.	Ubolsakka-Jones, C., Sangthong, B., Khrisanapant, W. *et al.*	The effect of slow-loaded breathing training on the blood pressure response to handgrip exercise in patients with isolated systolic hypertension.	*Hypertens Res* **40,** 885–891 (2017). https://doi.org/10.1038/hr.2017.54
71.	Grodin JL, Gallup D, Anstrom KJ, Felker GM, Chen HH, Tang WHW.	Implications of Alternative Hepatorenal Prognostic Scoring Systems in Acute Heart Failure (from DOSE-AHF and ROSE-AHF).	Am J Cardiol. 2017 Jun 15;119(12):2003-2009. doi: 10.1016/j.amjcard.2017.03.031. Epub 2017 Mar 29. PMID: 28433216; PMCID: PMC5477997.
72.	Rossum AC, Kramer MHH, Lammertsma AA, Knaapen P.	Chen WJY, Diamant M, de Boer K, Harms HJ, Robbers LFHJ, van Effects of exenatide on cardiac function, perfusion, and energetics in type 2 diabetic patients with cardiomyopathy: a randomized controlled trial against insulin glargine.	Cardiovasc Diabetol. 2017 May 19;16(1):67. doi: 10.1186/s12933-017-0549-z. PMID: 28526033; PMCID: PMC5438489.
73.	Bravo-Escobar R, González-Represas A, Gómez-González AM, Montiel-Trujillo A, Aguilar-Jimenez R, Carrasco-Ruíz R, Salinas-Sánchez P.	Effectiveness and safety of a home-based cardiac rehabilitation programme of mixed surveillance in patients with ischemic heart disease at moderate cardiovascular risk: A randomised, controlled clinical trial.	BMC Cardiovasc Disord. 2017 Feb 20;17(1):66. doi: 10.1186/s12872-017-0499-0. PMID: 28219338; PMCID: PMC5319164.
74.	Kirichenko TV, Myasoedova VA, Orekhova VA, Ravani AL, Nikitina NA, Grechko AV, Sobenin IA, Orekhov AN.	Phytoestrogen-Rich Natural Preparation for Treatment of Climacteric Syndrome and Atherosclerosis Prevention in Perimenopausal Women.	Phytother Res. 2017 Aug;31(8):1209-1214. doi: 10.1002/ptr.5841. Epub 2017 Jun 8. PMID: 28593720.
75.	Ghofrani HA, Simonneau G, D'Armini	Macitentan for the treatment of inoperable chronic thromboembolic pulmonary hypertension (MERIT-1): results from the	Lancet Respir Med. 2017 Oct;5(10):785-794. doi:

	AM, Fedullo P, Howard LS, Jaïs X, Jenkins DP, Jing ZC, Madani MM, Martin N, Mayer E, Papadakis K, Richard D, Kim NH; MERIT study investigators.	multicentre, phase 2, randomised, double-blind, placebo-controlled study.	10.1016/S2213-2600(17)30305-3. Epub 2017 Sep 11. PMID: 28919201.
76.	Houston TK, Fix GM, Shimada SL, Long JA, Gordon HS, Pope C, Volkman J, Allison JJ, DeLaughter K, Orner M, Bokhour BG.	African American Veterans Storytelling: A Multisite Randomized Trial to Improve Hypertension.	Med Care. 2017 Sep;55 Suppl 9 Suppl 2:S50-S58. doi: 10.1097/MLR.0000000000000766. PMID: 28806366.
77.	Nosaka K, Miyoshi T, Iwamoto M, Kajiya M, Okawa K, Tsukuda S, Yokohama F, Sogo M, Nishibe T, Matsuo N, Hirohata S, Ito H, Doi M.	Early initiation of eicosapentaenoic acid and statin treatment is associated with better clinical outcomes than statin alone in patients with acute coronary syndromes: 1-year outcomes of a randomized controlled study.	Int J Cardiol. 2017 Feb 1;228:173-179. doi: 10.1016/j.ijcard.2016.11.105. Epub 2016 Nov 9. PMID: 27865182.
78.	Baranova EV, Verhoef TI, Ragia G, le Cessie S, Asselbergs FW, de Boer A, Manolopoulos VG, Maitland-van der Zee AH;	EU-PACT group. Dosing algorithms for vitamin K antagonists across VKORC1 and CYP2C9 genotypes	. J Thromb Haemost. 2017 Mar;15(3):465-472. doi: 10.1111/jth.13615. Epub 2017 Feb 17. PMID: 28063245.

79.	Röger S, Said S, Kloppe A, Lawo T, Emig U, Rousso B, Gutterman D, Borggrefe M, Kuschyk J.	Cardiac contractility modulation in heart failure patients: Randomized comparison of signal delivery through one vs. two ventricular leads.	J Cardiol. 2017 Jan;69(1):326-332. doi: 10.1016/j.jjcc.2016.06.015. Epub 2016 Aug 30. PMID: 27590412.
80.	Saver JL, Carroll JD, Thaler DE, Smalling RW, MacDonald LA, Marks DS, Tirschwell DL;	RESPECT Investigators. Long-Term Outcomes of Patent Foramen Ovale Closure or Medical Therapy after Stroke.	N Engl J Med. 2017 Sep 14;377(11):1022-1032. doi: 10.1056/NEJMoa1610057. PMID: 28902590.
81.	Kwakernaak AJ, Roksnoer LC, Lambers Heerspink HJ, van den Berg-Garrelds I, Lochorn GA, van Embden Andres JH, Klijn MA, Kobori H, Danser AH, Laverman GD, Navis GJ.	Effects of Direct Renin Blockade on Renal & Systemic Hemodynamics and on RAAS Activity, in Weight Excess and Hypertension: A Randomized Clinical Trial.	PLoS One. 2017 Jan 24;12(1):e0169258. doi: 10.1371/journal.pone.0169258. PMID: 28118402; PMCID: PMC5261569.
82.	Moreira DM, Lueneberg ME, da Silva RL, Fattah T, Gottschall CAM.	MethotrexaTE THerapy in ST-Segment Elevation MYocardial InfarctionS: A Randomized Double-Blind, Placebo-Controlled Trial (TETHYS Trial).	J Cardiovasc Pharmacol Ther. 2017 Nov;22(6):538-545. doi: 10.1177/1074248417699884. Epub 2017 Mar 22. PMID: 28325070.
83.	Mizuno H, Hoshide S, Tomitani N, Kario K.	Comparison of ambulatory blood pressure-lowering effects of higher doses of different calcium antagonists in uncontrolled hypertension: the Calcium Antagonist Controlled-Release High-Dose Therapy in Uncontrolled Refractory Hypertensive Patients (CARILLON) Study.	Blood Press. 2017 Oct;26(5):284-293. doi: 10.1080/08037051.2017.1329623. Epub 2017 May 19. PMID: 28524699.

84.	Johnson SA, Feresin RG, Navaei N, Figueroa A, Elam ML, Akhavan NS, Hooshmand S, Pourafshar S, Payton ME, Arjmandi BH.	Effects of daily blueberry consumption on circulating biomarkers of oxidative stress, inflammation, and antioxidant defense in postmenopausal women with pre- and stage 1-hypertension: a randomized controlled trial.	Food Funct. 2017 Jan 25;8(1):372-380. doi: 10.1039/c6fo01216g. PMID: 28059417.
85.	Gunawardhana L, McLean L, Punzi HA, Hunt B, Palmer RN, Whelton A, Feig DI.	Effect of Febuxostat on Ambulatory Blood Pressure in Subjects With Hyperuricemia and Hypertension: A Phase 2 Randomized Placebo-Controlled Study.	J Am Heart Assoc. 2017 Nov 4;6(11):e006683. doi: 10.1161/JAHA.117.006683. PMID: 29102979; PMCID: PMC5721765.
86.	Kang HJ, Bae KY, Kim SW, Shin IS, Hong YJ, Ahn Y, Jeong MH, Yoon JS, Kim JM.	Effects of Escitalopram on Anxiety in Patients with Acute Coronary Syndrome: A Randomized Controlled Trial.	Clin Psychopharmacol Neurosci. 2017 May 31;15(2):126-131. doi: 10.9758/cpn.2017.15.2.126. PMID: 28449559; PMCID: PMC5426500.
87.	Body R, Boachie C, McConnachie A, Carley S, Van Den Berg P, Lecky FE.	Feasibility of the Manchester Acute Coronary Syndromes (MACS) decision rule to safely reduce unnecessary hospital admissions: a pilot randomised controlled trial.	Emerg Med J. 2017 Sep;34(9):586-592. doi: 10.1136/emermed-2016-206148. Epub 2017 May 12. PMID: 28500087; PMCID: PMC5574380.
88.	Brenner S, Christa M, Berliner D, Deubner N, Ertl G, Held M, Marx A, Angermann CE, Störk S, Rutten FH, Güder G.	Frequency and prognostic impact of mid-expiratory flow reduction in stable patients six months after hospitalisation for heart failure with reduced ejection fraction.	Int J Cardiol. 2017 Jan 15;227:727-733. doi: 10.1016/j.ijcard.2016.10.071. Epub 2016 Oct 28. PMID: 27816302.
89.	Sibbing D, Aradi D, Jacobshagen C, Gross L,	Guided de-escalation of antiplatelet treatment in patients with acute coronary syndrome undergoing percutaneous	Lancet. 2017 Oct 14;390(10104):1747-1757. doi: 10.1016/S0140-

	Trenk D, Geisler T, Orban M, Hadamitzky M, Merkely B, Kiss RG, Komócsi A, Dézsi CA, Holdt L, Felix SB, Parma R, Klopotowski M, Schwinger RHG, Rieber J, Huber K, Neumann FJ, Koltowski L, Mehilli J, Huczek Z, Massberg S; TROPICAL-ACS Investigators.v	coronary intervention (TROPICAL-ACS): a randomised, open-label, multicentre trial.	6736(17)32155-4. Epub 2017 Aug 28. PMID: 28855078.
90.	Ohman EM, Roe MT, Steg PG, James SK, Povsic TJ, White J, Rockhold F, Plotnikov A, Mundl H, Strony J, Sun X, Husted S, Tendera M, Montalescot G, Bahit MC, Ardissino D, Bueno H, Claeys MJ, Nicolau JC, Cornel JH, Goto S, Kiss RG, Güray Ü, Park DW, Bode C, Welsh RC, Gibson CM.	Clinically significant bleeding with low-dose rivaroxaban versus aspirin, in addition to P2Y12 inhibition, in acute coronary syndromes (GEMINI-ACS-1): a double-blind, multicentre, randomised trial.	Lancet. 2017 May 6;389(10081):1799-1808. doi: 10.1016/S0140-6736(17)30751-1. Epub 2017 Mar 18. PMID: 28325638.

91.	Jiang J, Cong H, Zhang Y, Li Z, Tao G, Li X, Qing L, Tan N, Zhao Z, Dong Y, Ji Z, Chen Y, Ge J, He B, Sun Y, Cao K, Huo Y.	Effect of Metoprolol Succinate in Patients with Stable Angina and Elevated Heart Rate Receiving Low-Dose β-Blocker Therapy.	Int J Med Sci. 2017 Apr 9;14(5):477-483. doi: 10.7150/ijms.18054. PMID: 28539824; PMCID: PMC5441040.
92.	Asher E, Frydman S, Katz M, Regev E, Sabbag A, Mazin I, Abu-Much A, Kukuy A, Mazo A, Erez A, Berkovitch A, Narodistky M, Barbash I, Segev A, Beigel R, Matetzky S.	Chewing versus Swallowing Ticagrelor to Accelerate Platelet Inhibition in Acute Coronary Syndrome - the CHEERS study. For The PLATIS (Platelets and Thrombosis in Sheba) Study Group.	Thromb Haemost. 2017 Apr 3;117(4):727-733. doi: 10.1160/TH16-09-0728. Epub 2017 Feb 2. PMID: 28150850.
93.	Tan BY, Shafie AA, Hassali MAA, Saleem F.	Assessment of medication adherence and the costs associated with a calendar blister pack intervention among hypertensive patients in Malaysia: A randomized controlled trial.	SAGE Open Med. 2017 Aug 3;5:2050312117709189. doi: 10.1177/2050312117709189. PMID: 28839933; PMCID: PMC5546697.
94.	Zhao Y, Peng R, Zhao W, Liu Q, Guo Y, Zhao S, Xu D.	Zhibitai and low-dose atorvastatin reduce blood lipids and inflammation in patients with coronary artery disease.	Medicine (Baltimore). 2017 Feb;96(7):e6104. doi: 10.1097/MD.0000000000006104. PMID: 28207527; PMCID: PMC5319516.
95.	Japaridze L, Sadunishvili M.	The short-term effect of atorvastatin plus ezetimibe therapy versus atorvastatin monotherapy on clinical outcome in acute coronary syndrome patients by gender.	Kardiol Pol. 2017;75(8):770-778. doi: 10.5603/KP.a2017.0074. Epub 2017 May 29. PMID: 28553847.
96.	Cherian TS, Shrader P,	Effect of Atrial Fibrillation on Mortality, Stroke Risk, and Quality-of-Life Scores in	Am J Cardiol. 2017 Jun

	Fonarow GC, Allen LA, Piccini JP, Peterson ED, Thomas L, Kowey PR, Gersh BJ, Mahaffey KW.	Patients With Heart Failure (from the Outcomes Registry for Better Informed Treatment of Atrial Fibrillation [ORBIT-AF]).	1;119(11):1763-1769. doi: 10.1016/j.amjcard.2017.02.050. Epub 2017 Mar 16. PMID: 28416199.
97.	Yano Y, Rakugi H, Bakris GL, Lloyd-Jones DM, Oparil S, Saruta T, Shimada K, Matsuoka H, Imai Y, Ogihara T.	On-Treatment Blood Pressure and Cardiovascular Outcomes in Older Adults With Isolated Systolic Hypertension.	Hypertension. 2017 Feb;69(2):220-227. doi: 10.1161/HYPERTENSIONAHA.116.08600. Epub 2017 Jan 3. PMID: 28049699.
98.	Wan Y, Li L, Niu H, Ma X, Yang J, Yuan C, Mu G, Zhang J.	Impact of Compound Hypertonic Saline Solution on Decompensated Heart Failure.	Int Heart J. 2017 Aug 3;58(4):601-607. doi: 10.1536/ihj.16-313. Epub 2017 Jul 13. PMID: 28701670. China, English
99.	Fanola CL, Morrow DA, Cannon CP, Jarolim P, Lukas MA, Bode C, Hochman JS, Goodrich EL, Braunwald E, O'Donoghue ML.	Interleukin-6 and the Risk of Adverse Outcomes in Patients After an Acute Coronary Syndrome: Observations From the SOLID-TIMI 52 (Stabilization of Plaque Using Darapladib-Thrombolysis in Myocardial Infarction 52) Trial.	J Am Heart Assoc. 2017 Oct 24;6(10):e005637. doi: 10.1161/JAHA.117.005637. PMID: 29066436; PMCID: PMC5721825.
100.	Volpp KG, Troxel AB, Mehta SJ, Norton L, Zhu J, Lim R, Wang W, Marcus N, Terwiesch C, Caldarella K, Levin T, Relish M, Negin N, Smith-McLallen	Effect of Electronic Reminders, Financial Incentives, and Social Support on Outcomes After Myocardial Infarction: The HeartStrong Randomized Clinical Trial.	JAMA Intern Med. 2017 Aug 1;177(8):1093-1101. doi: 10.1001/jamainternmed.2017.2449. PMID: 28654972; PMCID: PMC5710431.

	A, Snyder R, Spettell CM, Drachman B, Kolansky D, Asch DA.		
101.	Hua CY, Huang Y, Su YH, Bu JY, Tao HM.	Collaborative care model improves self-care ability, quality of life and cardiac function of patients with chronic heart failure.	Braz J Med Biol Res. 2017 Sep 21;50(11):e6355. doi: 10.1590/1414-431X20176355. PMID: 28953989; PMCID: PMC5609602.
102.	Prior DL, Stevens SR, Holly TA, Krejca M, Paraforos A, Pohost GM, Byrd K, Kukulski T, Jones RH, Desvigne-Nickens P, Varadarajan P, Amanullah A, Lin G, Al-Khalidi HR, Aldea G, Santambrogio C, Bochenek A, Berman DS; STICH Trial Investigators.	Regional left ventricular function does not predict survival in ischaemic cardiomyopathy after cardiac surgery.	Heart. 2017 Sep;103(17):1359-1367. doi: 10.1136/heartjnl-2016-310693. Epub 2017 Apr 26. PMID: 28446548; PMCID: PMC5564397.
103.	Gebhard C, Rhéaume E, Berry C, Brand G, Kernaleguen AE, et al. (2017)	Beneficial Effects of Reconstituted High-Density Lipoprotein (rHDL) on Circulating $CD34^+$ Cells in Patients after an Acute Coronary Syndrome.	PLOS ONE 12(1): e0168448. https://doi.org/10.1371/journal.pone.0168448
104.	Hammoudi N, Laveau F, Helft G, Cozic N, Barthelemy O, Ceccaldi A, Petroni T, Berman	Low level exercise echocardiography helps diagnose early stage heart failure with preserved ejection fraction: a study of echocardiography versus catheterization.	Clin Res Cardiol. 2017 Mar;106(3):192-201. doi: 10.1007/s00392-016-1039-0. Epub 2016 Sep 30. PMID: 27695989.

	E, Komajda M, Michel PL, Mallet A, Le Feuvre C, Isnard R.		
105.	Arturi F, Succurro E, Miceli S, Cloro C, Ruffo M, Maio R, Perticone M, Sesti G, Perticone F.	Liraglutide improves cardiac function in patients with type 2 diabetes and chronic heart failure.	Endocrine. 2017 Sep;57(3):464-473. doi: 10.1007/s12020-016-1166-4. Epub 2016 Nov 9. PMID: 27830456.
106.	Galiè N, Grimminger F, Grünig E, Hoeper MM, Humbert M, Jing ZC, Keogh AM, Langleben D, Rubin LJ, Fritsch A, Davie N, Ghofrani HA.	Comparison of hemodynamic parameters in treatment-naïve and pre-treated patients with pulmonary arterial hypertension in the randomized phase III PATENT-1 study.	J Heart Lung Transplant. 2017 May;36(5):509-519. doi: 10.1016/j.healun.2016.12.012. Epub 2016 Dec 24. PMID: 28190787.
107.	David E,	Randomized comparison of Ridaforolimus- and Zotarolimus -Eluting Coronary Stents in Patients with Coronary Artery Disease ,	Circulation, 2017;136;1304-1314, USA , English
108.	Hirai DM, Zelt JT, Jones JH, Castanhas LG, Bentley RF, Earle W, Staples P, Tschakovsky ME, McCans J, O'Donnell DE, Neder JA.	Dietary nitrate supplementation and exercise tolerance in patients with heart failure with reduced ejection fraction.	Am J Physiol Regul Integr Comp Physiol. 2017 Jan 1;312(1):R13-R22. doi: 10.1152/ajpregu.00263.2016. Epub 2016 Oct 26. PMID: 27784687.
109.	Grassi G, Seravalle G, Brambilla G, Dell'Oro R, Trevano FQ, Fici F, van Bortel L, Mancia G.	Multicenter Randomized Double-Blind Comparison of Nebivolol plus HCTZ and Irbesartan plus HCTZ in the Treatment of Isolated Systolic Hypertension in Elderly Patients: Results of the NEHIS Study.	Adv Ther. 2017 Jan;33(12):2173-2187. doi: 10.1007/s12325-016-0427-1. Epub 2016 Oct 22. PMID: 27771847.

110.	Maldonado-Martín S, Brubaker PH, Eggebeen J, Stewart KP, Kitzman DW.	Association Between 6-Minute Walk Test Distance and Objective Variables of Functional Capacity After Exercise Training in Elderly Heart Failure Patients With Preserved Ejection Fraction: A Randomized Exercise Trial.	Arch Phys Med Rehabil. 2017 Mar;98(3):600-603. doi: 10.1016/j.apmr.2016.08.481. Epub 2016 Sep 28. PMID: 27693420; PMCID: PMC7707852.
111.	Bangalore S, Davis BR, Cushman WC, Pressel SL, Muntner PM, Calhoun DA, Kostis JB, Whelton PK, Probstfield JL, Rahman M, Black HR; ALLHAT Collaborative	Research Group. Treatment-Resistant Hypertension and Outcomes Based on Randomized Treatment Group in ALLHAT.	Am J Med. 2017 Apr;130(4):439-448.e9. doi: 10.1016/j.amjmed.2016.10.002. Epub 2016 Oct 27. PMID: 27984005; PMCID: PMC5362319.
112.	Lindholm D, Hagström E, James SK, Becker RC, Cannon CP, Himmelmann A, Katus HA, Maurer G, López-Sendón JL, Steg PG, Storey RF, Siegbahn A, Wallentin L	. Growth Differentiation Factor 15 at 1 Month After an Acute Coronary Syndrome Is Associated With Increased Risk of Major Bleeding.	J Am Heart Assoc. 2017 Apr 14;6(4):e005580. doi: 10.1161/JAHA.117.005580. PMID: 28411246; PMCID: PMC5533037.
113.	Perez A, Cao C.	Azilsartan in Patients With Mild to Moderate Hypertension Using Clinic and Ambulatory Blood Pressure Measurements.	J Clin Hypertens (Greenwich). 2017 Jan;19(1):82-89. doi: 10.1111/jch.12873. Epub 2016 Jul 15. PMID: 27421242; PMCID: PMC8030785.
114.	Ouweneel DM, Eriksen E,	Percutaneous Mechanical Circulatory Support Versus Intra-Aortic Balloon	J Am Coll Cardiol. 2017 Jan 24;69(3):278-287.

	Sjauw KD, van Dongen IM, Hirsch A, Packer EJ, Vis MM, Wykrzykowska JJ, Koch KT, Baan J, de Winter RJ, Piek JJ, Lagrand WK, de Mol BA, Tijssen JG, Henriques JP.	Pump in Cardiogenic Shock After Acute Myocardial Infarction.	doi: 10.1016/j.jacc.2016.10.022. Epub 2016 Oct 31. PMID: 27810347.
115.	Snipelisky D, Kelly J, Levine JA, Koepp GA, Anstrom KJ, McNulty SE, Zakeri R, Felker GM, Hernandez AF, Braunwald E, Redfield MM.	Accelerometer-Measured Daily Activity in Heart Failure With Preserved Ejection Fraction: Clinical Correlates and Association With Standard Heart Failure Severity Indices.; PMCID: PMC5634329.	Circ Heart Fail. 2017 Jun;10(6):e003878. doi: 10.1161/CIRCHEARTFAILURE.117.003878. PMID: 28588021
116.	Miao ZL, Hou AJ, Zang HY, Huang RG, Zheng XQ, Lin HL, Wang W, Hou P, Xia F, Li ZQ.	Effects of recombinant human brain natriuretic peptide on the prognosis of patients with acute anterior myocardial infarction undergoing primary percutaneous coronary intervention: a prospective, multi-center, randomized clinical trial.	J Thorac Dis. 2017 Jan;9(1):54-63. doi: 10.21037/jtd.2017.01.15. PMID: 28203406; PMCID: PMC5303080.
117.	Sherwood A, Blumenthal JA, Koch GG, Hoffman BM, Watkins LL, Smith PJ, O'Connor CM, Adams KF Jr, Rogers JG, Sueta C, Chang PP,	Effects of Coping Skills Training on Quality of Life, Disease Biomarkers, and Clinical Outcomes in Patients With Heart Failure: A Randomized Clinical Trial.	Circ Heart Fail. 2017 Jan;10(1):e003410. doi: 10.1161/CIRCHEARTFAILURE.116.003410. PMID: 28062537; PMCID: PMC5233412.

	Johnson KS, Schwartz J, Hinderliter AL.		
118.	Chung MJ, Novak E, Brown DL.	Effect of prompt revascularization on outcomes in diabetic patients with stable ischemic heart disease and previous myocardial infarction in the Bypass Angioplasty Revascularization Investigation 2 Diabetes (BARI 2D) trial.	Coron Artery Dis. 2017 Jun;28(4):301-306. doi: 10.1097/MCA.0000000000000492. PMID: 28346285.
119.	Skaf S, Thibault B, Khairy P, O'Meara E, Fortier A, Vakulenko HV, Pitre C, White M, Ducharme A; EARTH Investigators.	Impact of Left Ventricular vs Biventricular Pacing on Reverse Remodelling: Insights From the Evaluation of Resynchronization Therapy for Heart Failure (EARTH) Trial.	Can J Cardiol. 2017 Oct;33(10):1274-1282. doi: 10.1016/j.cjca.2017.07.478. Epub 2017 Jul 31. PMID: 28941607.
120.	Petersen LA, Ramos KS, Pietz K, Woodard LD.	Impact of a Pay-for-Performance Program on Care for Black Patients with Hypertension: Important Answers in the Era of the Affordable Care Act.	Health Serv Res. 2017 Jun;52(3):1138-1155. doi: 10.1111/1475-6773.12517. Epub 2016 Jun 22. PMID: 27329344; PMCID: PMC5441487.
121.	Engeli S, May M, Nussberger J, Danser AHJ, Dole WP, Prescott MF, Dahlke M, Stitah S, Pal P, Boschmann M, Jordan J.	Systemic and tissue-specific effects of aliskiren on the RAAS and carbohydrate/lipid metabolism in obese patients with hypertension.	J Am Soc Hypertens. 2017 Aug;11(8):488-497. doi: 10.1016/j.jash.2017.06.002. Epub 2017 Jun 12. PMID: 28666704.
122.	Maldonado-Martín S, Brubaker PH, Eggebeen J, Stewart KP, Kitzman DW.	Association Between 6-Minute Walk Test Distance and Objective Variables of Functional Capacity After Exercise Training in Elderly Heart Failure Patients With Preserved Ejection Fraction: A Randomized Exercise Trial..	Arch Phys Med Rehabil. 2017 Mar;98(3):600-603. doi: 10.1016/j.apmr.2016.08.481. Epub 2016 Sep 28. PMID: 27693420; PMCID: PMC7707852

123.	Karjalainen P, Paana T, Ylitalo A, Sia J, Nammas W.	Optical coherence tomography follow-up 18 months after titanium-nitride-oxide-coated versus everolimus-eluting stent implantation in patients with acute coronary syndrome.	Acta Radiol. 2017 Sep;58(9):1077-1084. doi: 10.1177/0284185116683573. Epub 2016 Dec 19. PMID: 28273737.
124.	Minneboo M, Lachman S, Snaterse M, Jørstad HT, Ter Riet G, Boekholdt SM, Scholte Op Reimer WJM, Peters RJG;	RESPONSE-2 Study Group. Community-Based Lifestyle Intervention in Patients With Coronary Artery Disease: The RESPONSE-2 Trial.	J Am Coll Cardiol. 2017 Jul 18;70(3):318-327. doi: 10.1016/j.jacc.2017.05.041. PMID: 28705312.
125.	Grejs AM, Nielsen BRR, Juhl-Olsen P, Gjedsted J, Sloth E, Heiberg J, Frederiksen CA, Jeppesen AN, Duez CHV, Hamre PD, Søreide E, Kirkegaard H.	Effect of prolonged targeted temperature management on left ventricular myocardial function after out-of-hospital cardiac arrest - A randomised, controlled trial.	Resuscitation. 2017 Jun;115:23-31. doi: 10.1016/j.resuscitation.2017.03.021. Epub 2017 Apr 2. PMID: 28377297.
126.	Mende CW, Giles TD, Bharucha DB, Ferguson WG, Mallick M, Patel MD.	Efficacy of nebivolol-valsartan single-pill combination in obese and nonobese patients with hypertension.	J Clin Hypertens (Greenwich). 2017 Jun;19(6):632-639. doi: 10.1111/jch.12965. Epub 2017 Jan 11. PMID: 28075064; PMCID: PMC5484387.
127.	Liu, Y., Kong, X., Wang, W., Fan, F., Zhang, Y., Zhao, M., Wang, Y., Wang, Y., Wang, Y., Qin, X., Tang, G., Wang, B.,	Association of peripheral differential leukocyte counts with dyslipidemia risk in Chinese patients with hypertension: insight from the China Stroke Primary Prevention Trial.	*Journal of lipid research*, *58*(1), 256–266. https://doi.org/10.1194/jlr.P067686

	Xu, X., Hou, F. F., Gao, W., Sun, N., Li, J., Venners, S. A., Jiang, S., & Huo, Y. (2017).		
128.	Pu J, Ding S, Ge H, Han Y, Guo J, Lin R, Su X, Zhang H, Chen L, He B; EARLY-MYO Investigators.	Efficacy and Safety of a Pharmaco-Invasive Strategy With Half-Dose Alteplase Versus Primary Angioplasty in ST-Segment-Elevation Myocardial Infarction: EARLY-MYO Trial (Early Routine Catheterization After Alteplase Fibrinolysis Versus Primary PCI in Acute ST-Segment-Elevation Myocardial Infarction).	Circulation. 2017 Oct 17;136(16):1462-1473. doi: 10.1161/CIRCULATIONAHA.117.030582. Epub 2017 Aug 27. Erratum in: Circulation. 2018 Feb 13;137(7):e29. PMID: 28844990.
129.	Upadhya B, Rocco M, Lewis CE, Oparil S, Lovato LC, Cushman WC, Bates JT, Bello NA, Aurigemma G, Fine LJ, Johnson KC, Rodriguez CJ, Raj DS, Rastogi A, Tamariz L, Wiggers A, Kitzman DW; SPRINT Research Group.	Effect of Intensive Blood Pressure Treatment on Heart Failure Events in the Systolic Blood Pressure Reduction Intervention Trial.	Circ Heart Fail. 2017 Apr;10(4):e003613. doi: 10.1161/CIRCHEARTFAILURE.116.003613. PMID: 28364091; PMCID: PMC5384646.
130.	Flintholm Raft K, Frestad D, Michelsen MM, Suhrs HE, Rask AB, Nilsson M, Hermann TS, Prescott E.	Peripheral Endothelial Function and Coronary Flow Velocity Reserve Are Not Associated in Women with Angina and No Obstructive Coronary Artery Disease: The iPOWER Study.	J Vasc Res. 2017;54(5):309-319. doi: 10.1159/000479374. Epub 2017 Sep 23. PMID: 28942444.
131.	Grejs AM, Gjedsted J, Thygesen	The Extent of Myocardial Injury During Prolonged Targeted Temperature	Am J Med. 2017 Jan;130(1):37-46. doi:

	K, Lassen JF, Rasmussen BS, Jeppesen AN, Duez CH, Søreide E, Kirkegaard H.	Management After Out-of-Hospital Cardiac Arrest.	10.1016/j.amjmed.2016.06.047. Epub 2016 Jul 29. PMID: 27477668.
132.	Waldmann E, Vogt A, Crispin A, Altenhofer J, Riks I, Parhofer KG	. Effect of mipomersen on LDL-cholesterol in patients with severe LDL-hypercholesterolaemia and atherosclerosis treated by lipoprotein apheresis (The MICA-Study).	Atherosclerosis. 2017 Apr;259:20-25. doi: 10.1016/j.atherosclerosis.2017.02.019. Epub 2017 Feb 24. Erratum in: Atherosclerosis. 2018 Aug;275:461-462. PMID: 28279833.
133.	Goldstein SA, Newby LK, Cyr DD, Neely M, Lüscher TF, Brown EB, White HD, Ohman EM, Roe MT, Hamm CW.	Relationship Between Peak Troponin Values and Long-Term Ischemic Events Among Medically Managed Patients With Acute Coronary Syndromes	. J Am Heart Assoc. 2017 Apr 11;6(4):e005334. doi: 10.1161/JAHA.116.005334. PMID: 28400368; PMCID: PMC5533023.
134.	Kirkegaard H, Søreide E, de Haas I, Pettilä V, Taccone FS, Arus U, Storm C, Hassager C, Nielsen JF, Sørensen CA, Ilkjær S, Jeppesen AN, Grejs AM, Duez CHV, Hjort J, Larsen AI, Toome V, Tiainen M, Hästbacka J, Laitio T, Skrifvars MB.	Targeted Temperature Management for 48 vs 24 Hours and Neurologic Outcome After Out-of-Hospital Cardiac Arrest: A Randomized Clinical Trial.	JAMA. 2017 Jul 25;318(4):341-350. doi: 10.1001/jama.2017.8978. PMID: 28742911; PMCID: PMC5541324.

135.	Amara W, Montagnier C, Cheggour S, Boursier M, Gully C, Barnay C, Georger F, Deplagne A, Fromentin S, Mlotek M, Lazarus A, Taïeb J; SETAM Investigators.	Early Detection and Treatment of Atrial Arrhythmias Alleviates the Arrhythmic Burden in Paced Patients: The SETAM Study.	Pacing Clin Electrophysiol. 2017 May;40(5):527-536. doi: 10.1111/pace.13062. Epub 2017 Mar 23. PMID: 28244117.
136.	Li Y, Qin X, Luo L, Wang B, Huo Y, Hou FF, Xu X.	Folic acid therapy reduces the risk of mortality associated with heavy proteinuria among hypertensive patients.	J Hypertens. 2017 Jun;35(6):1302-1309. doi: 10.1097/HJH.0000000000001292. PMID: 28441699.
137.	Packer M, O'Connor C, McMurray JJV, Wittes J, Abraham WT, Anker SD, Dickstein K, Filippatos G, Holcomb R, Krum H, Maggioni AP, Mebazaa A, Peacock WF, Petrie MC, Ponikowski P, Ruschitzka F, van Veldhuisen DJ, Kowarski LS, Schactman M, Holzmeister J; TRUE-AHF Investigators.	Effect of Ularitide on Cardiovascular Mortality in Acute Heart Failure. N Engl	J Med. 2017 May 18;376(20):1956-1964. doi: 10.1056/NEJMoa1601895. Epub 2017 Apr 12. PMID: 28402745.

138.	Ter Maaten JM, Maggioni AP, Latini R, Masson S, Tognoni G, Tavazzi L, Signorini S, Voors AA, Damman K.	Clinical and prognostic value of spot urinary creatinine in chronic heart failure-An analysis from GISSI-HF.	Am Heart J. 2017 Jun;188:189-195. doi: 10.1016/j.ahj.2017.01.017. Epub 2017 Apr 4. PMID: 28577676.
139.	Xiaojing C, Yanfang L, Yanqing G, Fangfang C.	Thymopentin improves cardiac function in older patients with chronic heart failure.	Anatol J Cardiol. 2017 Jan;17(1):24-30. doi: 10.14744/AnatolJCardiol.2016.6692. Epub 2016 Aug 23. PMID: 27564775; PMCID: PMC5324858.
140.	CM, Chandiramani R, Huber K, Kornowski R, Weisz G, Kunadian V, Oldroyd KG, Ya-Ling H, Kaul U, Witzenbichler B, Dudek D, Sardella G, Escaned J, Sharma S, Shlofmitz RA, Collier T, Pocock S, Mehran R.	Angiolillo DJ, Baber U, Sartori S, Briguori C, Dangas G, Cohen DJ, Mehta SR, Gibson Ticagrelor With or Without Aspirin in High-Risk Patients With Diabetes Mellitus Undergoing Percutaneous Coronary Intervention.	J Am Coll Cardiol. 2020 May 19;75(19):2403-2413. doi: 10.1016/j.jacc.2020.03.008. Epub 2020 Mar 30. PMID: 32240760.
141.	Ballantyne CM, Shah S, Sapre A, Ashraf TB, Tobias SC, Sahin T, Ye P, Dong Y, Sheu WH, Kang DH, Ferreira Rossi PR, Moiseeva Y, Briones IR, Johnson-	Multiregional, Randomized Evaluation of the Lipid-Modifying Efficacy and Tolerability of Anacetrapib Added to Ongoing Statin Therapy in Patients With Hypercholesterolemia or Low High-Density Lipoprotein Cholesterol.	Am J Cardiol. 2017 Aug 15;120(4):569-576. doi: 10.1016/j.amjcard.2017.03.255. Epub 2017 Apr 12. PMID: 28624096.

	Levonas AO, Mitchel YB. A		
142.	Mogensen UM, Jhund PS, Abraham WT, Desai AS, Dickstein K, Packer M, Rouleau JL, Solomon SD, Swedberg K, Zile MR, Køber L, McMurray JJV; PARADIGM-HF and ATMOSPHERE Investigators and Committees.	Type of Atrial Fibrillation and Outcomes in Patients With Heart Failure and Reduced Ejection Fraction.	J Am Coll Cardiol. 2017 Nov 14;70(20):2490-2500. doi: 10.1016/j.jacc.2017.09.027. PMID: 29145948.
143.	Elming MB, Nielsen JC, Haarbo J, Videbæk L, Korup E, Signorovitch J, Olesen LL, Hildebrandt P, Steffensen FH, Bruun NE, Eiskjær H, Brandes A, Thøgersen AM, Gustafsson F, Egstrup K, Videbæk R, Hassager C, Svendsen JH, Høfsten DE, Torp-Pedersen C, Pehrson S, Køber L, Thune JJ.	Age and Outcomes of Primary Prevention Implantable Cardioverter-Defibrillators in Patients With Nonischemic Systolic Heart Failure.	Circulation. 2017 Nov 7;136(19):1772-1780. doi: 10.1161/CIRCULATIONAHA.117.028829. Epub 2017 Sep 6. PMID: 28877914.
144.	• M. Da	Prognostic Impact of Diabetes and Prediabetes on Survival Outcomes in	Journal of the American Heart

	• uriz, G. Targher, + 817 authors G. Sanna<br>• Published 1 July 2017	Patients With Chronic Heart Failure: A Post-Hoc Analysis of the GISSI-HF (Gruppo Italiano per lo Studio della Sopravvivenza nella Insufficienza Cardiaca-Heart Failure) Trial	Association: Cardiovascular and Cerebrovascular Disease
145.	Kawasaki M, Yamada T, Okuyama Y, Morita T, Furukawa Y, Tamaki S, Iwasaki Y, Kikuchi A, Sakata Y, Fukunami M.	Eplerenone might affect atrial fibrosis in patients with hypertension.	Pacing Clin Electrophysiol. 2017 Oct;40(10):1096-1102. doi: 10.1111/pace.13169. Epub 2017 Sep 23. PMID: 28845908.
146.	Hilfiker-Kleiner D, Haghikia A, Berliner D, Vogel-Claussen J, Schwab J, Franke A, Schwarzkopf M, Ehlermann P, Pfister R, Michels G, Westenfeld R, Stangl V, Kindermann I, Kühl U, Angermann CE, Schlitt A, Fischer D,	. Bromocriptine for the treatment of peripartum cardiomyopathy: a multicentre randomized study.	Eur Heart J. 2017 Sep 14;38(35):2671-2679. doi: 10.1093/eurheartj/ehx355. PMID: 28934837; PMCID: PMC5837241.

	Podewski E, Böhm M, Sliwa K, Bauersachs J		
147.	Pieske B, Maggioni AP, Lam CSP, Pieske-Kraigher E, Filippatos G, Butler J, Ponikowski P, Shah SJ, Solomon SD, Scalise AV, Mueller K, Roessig L, Gheorghiade M.	Vericiguat in patients with worsening chronic heart failure and preserved ejection fraction: results of the SOluble guanylate Cyclase stimulatoR in heArT failurE patientS with PRESERVED EF (SOCRATES-PRESERVED) study.	Eur Heart J. 2017 Apr 14;38(15):1119-1127. doi: 10.1093/eurheartj/ehw593. PMID: 28369340; PMCID: PMC5400074.
148.	Poreba M, Mostowik M, Siniarski A, Golebiowska-Wiatrak R, Malinowski KP, Haberka M, Konduracka E, Nessler J, Undas A, Gajos G.	Treatment with high-dose n-3 PUFAs has no effect on platelet function, coagulation, metabolic status or inflammation in patients with atherosclerosis and type 2 diabetes.	Cardiovasc Diabetol. 2017 Apr 14;16(1):50. doi: 10.1186/s12933-017-0523-9. PMID: 28410617; PMCID: PMC5391604.
149.	Grodin JL, Sun JL, Anstrom KJ, Chen HH, Starling RC, Testani JM, Tang WH.	Implications of Serum Chloride Homeostasis in Acute Heart Failure (from ROSE-AHF).	Am J Cardiol. 2017 Jan 1;119(1):78-83. doi: 10.1016/j.amjcard.2016.09.014. Epub 2016 Sep 30. PMID: 27816115; PMCID: PMC5161696.
150.	Goni L, Qi L, Cuervo M, Milagro FI, Saris WH, MacDonald IA, Langin D, Astrup A, Arner P, Oppert JM, Svendstrup	Effect of the interaction between diet composition and the *PPM1K* genetic variant on insulin resistance and β cell function markers during weight loss: results from the Nutrient Gene Interactions in Human Obesity: implications for dietary guidelines (NUGENOB) randomized trial.	Am J Clin Nutr. 2017 Sep;106(3):902-908. doi: 10.3945/ajcn.117.156281. Epub 2017 Aug 2. PMID: 28768654.

	M, Blaak EE, Sørensen TI, Hansen T, Martínez JA.		
151.	Hoedemaker NPG, Damman P, Woudstra P, Hirsch A, Windhausen F, Tijssen JGP, de Winter RJ; ICTUS Investigators.	Early Invasive Versus Selective Strategy for Non-ST-Segment Elevation Acute Coronary Syndrome: The ICTUS Trial.	J Am Coll Cardiol. 2017 Apr 18;69(15):1883-1893. doi: 10.1016/j.jacc.2017.02.023. PMID: 28408018.
152.	Johnson W, White WB, Sica D, Bakris GL, Weber MA, Handley A, Perez A, Cao C, Kupfer S, Saunders EB.	Evaluation of the angiotensin II receptor blocker azilsartan medoxomil in African-American patients with hypertension.	J Clin Hypertens (Greenwich). 2017 Jul;19(7):695-701. doi: 10.1111/jch.12993. Epub 2017 May 11. PMID: 28493376; PMCID: PMC8031359.
153.	Hong SJ, Jeong HS, Han SH, Chang KY, Hong BK, Lee BK, Chae SC, Kim WS, Park CG, Heo JH, Lee SU, Kim YD, Kim KS, Choi JH, Kang HJ, Kim JJ, Kang SM, Choi YJ, Shin JH, Chun KJ, Shin DG, Park SH, Kwan J, Choi YJ, Jeong MH, Chae JK, Kim DW, Cho JR,	Comparison of Fixed-dose Combinations of Amlodipine/Losartan Potassium/Chlorthalidone and Amlodipine/Losartan Potassium in Patients With Stage 2 Hypertension Inadequately Controlled With Amlodipine/Losartan Potassium: A Randomized, Double-blind, Multicenter, Phase III Study.	Clin Ther. 2017 Oct;39(10):2049-2060. doi: 10.1016/j.clinthera.2017.08.013. Epub 2017 Sep 19. PMID: 28939406.

	Han KR, Won KH, Park SH, Lee SK, Kim SH, Jung J, Kim CH.		
154.	Lee DH, Chun EJ, Hur JH, Min SH, Lee JE, Oh TJ, Kim KM, Jang HC, Han SJ, Kang DK, Kim HJ, Lim S.	Effect of sarpogrelate, a selective 5-HT_{2A} receptor antagonist, on characteristics of coronary artery disease in patients with type 2 diabetes.	Atherosclerosis. 2017 Feb;257:47-54. doi: 10.1016/j.atherosclerosis.2016.12.011. Epub 2016 Dec 10. PMID: 28068560.
155.	Moscarella E, Spitaleri G, Brugaletta S, Sentí Farrarons S, Pernigotti A, Ortega-Paz L, Cequier A, Iñiguez A, Serra A, Jiménez-Quevedo P, Mainar V, Campo G, Tespili M, den Heijer P, Bethencourt A, Vazquez N, Valgimigli M, Serruys PW, Sabaté M.	Impact of Body Mass Index on 5-Year Clinical Outcomes in Patients With ST-Segment Elevation Myocardial Infarction After Everolimus-Eluting or Bare-Metal Stent Implantation.	Am J Cardiol. 2017 Nov 1;120(9):1460-1466. doi: 10.1016/j.amjcard.2017.07.040. Epub 2017 Jul 31. PMID: 28864322.
156.	Pierre Deharo, MD , Gregory Ducrocq, MD, PhD, Christoph Bode, MD, Marc Cohen, MD, Thomas Cuisset, MD ,	Timing of Angiography and Outcomes in High-Risk Patients With Non–ST-Segment–Elevation Myocardial Infarction Managed Invasively Insights From the TAO Trial (Treatment of Acute Coronary Syndrome With Otamixaban	https://doi.org/10.1161/CIRCULATIONAHA.117.029779 Circulation. 2017;136:1895–1907

	PhD, Shamir R. Mehta, MD, Charles Pollack, Jr, MA, MD, Stephen D. Wiviott, MD, Yedid Elbez, MS, Marc S. Sabatine, MD, MPH, and Philippe Gabriel Steg, MD		
157.	Dasgupta K, Rosenberg E, Joseph L, Cooke AB, Trudeau L, Bacon SL, Chan D, Sherman M, Rabasa-Lhoret R, Daskalopoulou SS; SMARTER Trial Group.	Physician step prescription and monitoring to improve ARTERial health (SMARTER): A randomized controlled trial in patients with type 2 diabetes and hypertension.	Diabetes Obes Metab. 2017 May;19(5):695-704. doi: 10.1111/dom.12874. Epub 2017 Feb 22. PMID: 28074635; PMCID: PMC5412851.
158.	Çavuşoğlu Y, Zoghi M, Eren M, Bozçalı E, Kozdağ G, Şentürk T, Alicik G, Soylu K, Sarı İ, Berilgen R, Temizhan A, Gencer E, Orhan AL, Polat V, Aydın Kaderli A, Aktoz M, Zengin H, Aksoy M, Selçuk MT, Ergene O, Soran Ö; Hit-PoinT Investigators.	Post-discharge heart failure monitoring program in Turkey: Hit-PoinT.	Anatol J Cardiol. 2017 Feb;17(2):107-112. doi: 10.14744/AnatolJCardiol.2016.6812. Epub 2016 Jul 26. PMID: 27488754; PMCID: PMC5336747.

159.	Venetsanos D, Sederholm Lawesson S, Alfredsson J, *et al*	Association between gender and short-term outcome in patients with ST elevation myocardial infraction participating in the international, prospective, randomised Administration of Ticagrelor in the catheterisation Laboratory or in the Ambulance for New ST elevation myocardial Infarction to open the Coronary artery (ATLANTIC) trial: a prespecified analysis	*BMJ Open* 2017;**7:**e015241. doi: 10.1136/bmjopen-2016-015241
160.	Wojakowski W, Jadczyk T, Michalewska-Włudarczyk A, Parma Z, Markiewicz M, Rychlik W, Kostkiewicz M, Gruszczyńska K, Błach A, Dzier Zak-Mietła M, Wańha W, Ciosek J, Ochała B, Rzeszutko Ł, Cybulski W, Partyka Ł, Zasada W, Włudarczyk W, Dworowy S, Kuczmik W, Smolka G, Pawłowski T, Ochała A, Tendera M.	Effects of Transendocardial Delivery of Bone Marrow-Derived $CD133^{+}$ Cells on Left Ventricle Perfusion and Function in Patients With Refractory Angina: Final Results of Randomized, Double-Blinded, Placebo-Controlled REGENT-VSEL Trial.	Circ Res. 2017 Feb 17;120(4):670-680. doi: 10.1161/CIRCRESAHA.116.309009. Epub 2016 Nov 30. PMID: 27903568.
161.	Brener SJ, Mehran R, Dangas GD, Ohman EM, Witzenbichler B, Zhang Y, Parvataneni R, Stone GW.	Relation of Baseline Hemoglobin Levels and Adverse Events in Patients With Acute Coronary Syndromes (from the Acute Catheterization and Urgent Intervention Triage strategY and Harmonizing Outcomes with RevasculariZatiON and Stents in Acute Myocardial Infarction Trials).	Am J Cardiol. 2017 Jun 1;119(11):1710-1716. doi: 10.1016/j.amjcard.2017.02.052. Epub 2017 Mar 16. PMID: 28388994.

162.	Skielboe AK, Bandholm TQ, Hakmann S, Mourier M, Kallemose T, Dixen U.	Cardiovascular exercise and burden of arrhythmia in patients with atrial fibrillation - A randomized controlled trial.	PLoS One. 2017 Feb 23;12(2):e0170060. doi: 10.1371/journal.pone.0170060. PMID: 28231325; PMCID: PMC5322948.
163.	Felker GM, Anstrom KJ, Adams KF, Ezekowitz JA, Fiuzat M, Houston-Miller N, Januzzi JL Jr, Mark DB, Piña IL, Passmore G, Whellan DJ, Yang H, Cooper LS, Leifer ES, Desvigne-Nickens P, O'Connor CM.	Effect of Natriuretic Peptide-Guided Therapy on Hospitalization or Cardiovascular Mortality in High-Risk Patients With Heart Failure and Reduced Ejection Fraction: A Randomized Clinical Trial.	JAMA. 2017 Aug 22;318(8):713-720. doi: 10.1001/jama.2017.10565. PMID: 28829876; PMCID: PMC5605776.
164.	Felker GM, Anstrom KJ, Adams KF, Ezekowitz JA, Fiuzat M, Houston-Miller N, Januzzi JL Jr, Mark DB, Piña IL, Passmore G, Whellan DJ, Yang H, Cooper LS, Leifer ES, Desvigne-Nickens P, O'Connor CM.	Effect of Natriuretic Peptide-Guided Therapy on Hospitalization or Cardiovascular Mortality in High-Risk Patients With Heart Failure and Reduced Ejection Fraction: A Randomized Clinical Trial.	JAMA. 2017 Aug 22;318(8):713-720. doi: 10.1001/jama.2017.10565. PMID: 28829876; PMCID: PMC5605776.
165.	Fernandes-Silva MM, Guimarães GV, Rigaud	Inflammatory biomarkers and effect of exercise on functional capacity in patients with heart failure: Insights from a randomized clinical trial.	Eur J Prev Cardiol. 2017 May;24(8):808-817. doi:

	VO, Lofrano-Alves MS, Castro RE, de Barros Cruz LG, Bocchi EA, Bacal F.		10.1177/2047487317690458. Epub 2017 Jan 30. PMID: 28134562.
166.	Williams MC, Hunter A, Shah A, Assi V, Lewis S, Mangion K, Berry C, Boon NA, Clark E, Flather M, Forbes J, McLean S, Roditi G, van Beek EJ, Timmis AD, Newby DE; Scottish COmputed Tomography of the HEART (SCOT-HEART) Trial Investigators.	Symptoms and quality of life in patients with suspected angina undergoing CT coronary angiography: a randomised controlled trial.	Heart. 2017 Jul;103(13):995-1001. doi: 10.1136/heartjnl-2016-310129. Epub 2017 Feb 28. PMID: 28246175; PMCID: PMC5529983.
167.	Sun G, Liu F, Qu R.	Effect of High Thoracic Sympathetic Nerve Block on Serum Collagen Biomarkers in Patients with Chronic Heart Failure.	Cardiology. 2017;136(2):102-107. doi: 10.1159/000448165. Epub 2016 Sep 3. PMID: 27591776.
168.	Shi C, Men L, Yu C, Yao J, Bai R, Yang Y, Sun L, Sun G, Song G, Zhang Y, Xing Q, Du J.	Atherosclerosis associated with dynamic inflammation changes after multifactorial intervention in short-duration type 2 diabetes: A randomized, controlled, 10-year follow-up trial.	J Diabetes Complications. 2017 Aug;31(8):1286-1292. doi: 10.1016/j.jdiacomp.2017.05.008. Epub 2017 May 25. PMID: 28610945.
169.	Tani S, Nagao K, Yagi T, Atsumi W, Hirayama A.	Impact of Adding Eicosapentaenoic Acid to Statin Therapy on Plasma Pentraxin 3 Level in Patients with Stable Coronary Artery Disease: A 6-Month, Randomized Controlled Study.	Am J Cardiovasc Drugs. 2017 Feb;17(1):49-59. doi: 10.1007/s40256-016-0195-y. PMID: 27778191.

170.	Zou P, Dennis CL, Lee R, Parry M.	Hypertension Prevalence, Health Service Utilization, and Participant Satisfaction: Findings From a Pilot Randomized Controlled Trial in Aged Chinese Canadians.	Inquiry. 2017 Jan 1;54:46958017724942. doi: 10.1177/0046958017724942. PMID: 28853303; PMCID: PMC5798669.
171.	Chen Q, Shang X, Yuan M, Liang L, Zhong X.	Effect of atorvastatin on serum omentin-1 in patients with coronary artery disease.	Coron Artery Dis. 2017 Jan;28(1):44-51. doi: 10.1097/MCA.0000000000000435. PMID: 27749321.
172.	Cannon JA, Shen L, Jhund PS, Kristensen SL, Køber L, Chen F, Gong J, Lefkowitz MP, Rouleau JL, Shi VC, Swedberg K, Zile MR, Solomon SD, Packer M, McMurray JJ;	PARADIGM-HF Investigators and Committees. Dementia-related adverse events in PARADIGM-HF and other trials in heart failure with reduced ejection fraction.	Eur J Heart Fail. 2017 Jan;19(1):129-137. doi: 10.1002/ejhf.687. Epub 2016 Nov 20. PMID: 27868321; PMCID: PMC5248626.
173.	Masahiko Asami, Jiro Aoki, Shuzou Tanimoto, Yu Horiuchi, Mika Watanabe, Koichi Furui, Kentaro Yasuhara, Tatsuyuki Sato, Kengo Tanabe, Kazuhiro Hara,	Effects of Long-Acting Loop Diuretics in Heart Failure With Reduced Ejection Fraction Patients With Cardiac Resynchronization Therapy	, International Heart Journal, 2017, Volume 58, Issue 2, Pages 211-219, Released April 06, 2017, [Advance publication] Released March 17, 2017, Online ISSN 1349-3299, Print ISSN 1349-2365, https://doi.org/10.1536/ihj.16-290, https://www.jstage.jst.go.jp/article/ihj/58/2/58_16-290/_article/-char/en,
174.	Wang H, Anstrom K, Ilkayeva O, Muehlbauer MJ, Bain JR, McNulty S,	Sildenafil Treatment in Heart Failure With Preserved Ejection Fraction: Targeted Metabolomic Profiling in the RELAX Trial.	JAMA Cardiol. 2017 Aug 1;2(8):896-901. doi: 10.1001/jamacardio.2017.1239. PMID: 28492915; PMCID: PMC5815079.

	Newgard CB, Kraus WE, Hernandez A, Felker GM, Redfield M, Shah SH.		
175.	Erik Thunström, MD, PhD, Helena Glantz, MD, PhD, Tülay Yucel-Lindberg, PhD, Kristin Lindberg, MD, Mustafa Saygin, MD, PhD, Yüksel Peker, MD, PhD,	CPAP Does Not Reduce Inflammatory Biomarkers in Patients With Coronary Artery Disease and Nonsleepy Obstructive Sleep Apnea: A Randomized Controlled Trial,	*Sleep*, Volume 40, Issue 11, November 2017, zsx157, https://doi.org/10.1093/sleep/zsx157
176.	Casanova MA, Medeiros F, Trindade M, Cohen C, Oigman W, Neves MF.	Omega-3 fatty acids supplementation improves endothelial function and arterial stiffness in hypertensive patients with hypertriglyceridemia and high cardiovascular risk.	J Am Soc Hypertens. 2017 Jan;11(1):10-19. doi: 10.1016/j.jash.2016.10.004. Epub 2016 Oct 26. PMID: 27876342.
177.	Alenezi F, Brummett BH, Boyle SH, Samad Z, Babyak MA, Alzaeim N, Wilson J, Romano MMD, Sun JL, Ersboll M, O'Connor CM, Velazquez EJ, Jiang W.	Usefulness of Myocardial Annular Velocity Change During Mental Stress to Predict Cardiovascular Outcome in Patients With Coronary Artery Disease (From the Responses of Mental Stress-Induced Myocardial Ischemia to Escitalopram Treatment Trial).	Am J Cardiol. 2017 Nov 1;120(9):1495-1500. doi: 10.1016/j.amjcard.2017.07.039. Epub 2017 Jul 29. PMID: 28917493.
178.	Zheng J, Xiao T, Ye P, Miao D, Wu H.	Xuezhikang reduced arterial stiffness in patients with essential hypertension: a preliminary study.	Braz J Med Biol Res. 2017 Aug 31;50(10):e6363. doi: 10.1590/1414-431X20176363. PMID: 28876367;

			PMCID: PMC5579967.
179.	Mas JL, Derumeaux G, Guillon B, Massardier E, Hosseini H, Mechtouff L, Arquizan C, Béjot Y, Vuillier F, Detante O, Guidoux C, Canaple S, Vaduva C, Dequatre-Ponchelle N, Sibon I, Garnier P, Ferrier A, Timsit S, Robinet-Borgomano E, Sablot D, Lacour JC, Zuber M, Favrole P, Pinel JF, Apoil M, Reiner P, Lefebvre C, Guérin P, Piot C, Rossi R, Dubois-Randé JL, Eicher JC, Meneveau N, Lusson JR, Bertrand B, Schleich JM, Godart F, Thambo JB, Leborgne L, Michel P, Pierard L, Turc G, Barthelet M, Charles-Nelson A, Weimar C, Moulin T, Juliard JM,	Patent Foramen Ovale Closure or Anticoagulation vs. Antiplatelets after Stroke.	N Engl J Med. 2017 Sep 14;377(11):1011-1021. doi: 10.1056/NEJMoa1705915. PMID: 28902593.

	Chatellier G; CLOSE Investigators.		
180.	Buis L, Hirzel L, Dawood RM, Dawood KL, Nichols LP, Artinian NT, Schwiebert L, Yarandi HN, Roberson DN, Plegue MA, Mango LC, Levy PD.	Text Messaging to Improve Hypertension Medication Adherence in African Americans From Primary Care and Emergency Department Settings: Results From Two Randomized Feasibility Studies.	JMIR Mhealth Uhealth. 2017 Feb 1;5(2):e9. doi: 10.2196/mhealth.6630. PMID: 28148474; PMCID: PMC5311421.
181.	Lee CW, Kim M.	Effects of preanesthetic dexmedetomidine on hemodynamic responses to endotracheal intubation in elderly patients undergoing treatment for hypertension: a randomized, double-blinded trial.	Korean J Anesthesiol. 2017 Feb;70(1):39-45. doi: 10.4097/kjae.2017.70.1.39. Epub 2016 Dec 1. PMID: 28184265; PMCID: PMC5296386.
182.	O'Neal WT, Sandesara P, Patel N, Venkatesh S, Samman-Tahhan A, Hammadah M, Kelli HM, Soliman EZ.	Echocardiographic predictors of atrial fibrillation in patients with heart failure with preserved ejection fraction.	Eur Heart J Cardiovasc Imaging. 2017 Jul 1;18(7):725-729. doi: 10.1093/ehjci/jex038. PMID: 28379310; PMCID: PMC5837728.
183.	Ribaric SF, Turel M, Knafelj R, Gorjup V, Stanic R, Gradisek P, Cerovic O, Mirkovic T, Noc M.	Prophylactic versus clinically-driven antibiotics in comatose survivors of out-of-hospital cardiac arrest-A randomized pilot study.	Resuscitation. 2017 Feb;111:103-109. doi: 10.1016/j.resuscitation.2016.11.025. Epub 2016 Dec 14. PMID: 27987397.
184.	Ennis S, McGregor G, Hamborg T, Jones H, Shave R, Singh SJ, Banerjee P.	Randomised feasibility trial into the effects of low-frequency electrical muscle stimulation in advanced heart failure patients.	BMJ Open. 2017 Aug 11;7(8):e016148. doi: 10.1136/bmjopen-2017-016148. PMID: 28801415;

			PMCID: PMC5629639.
185.	de Oliveira PA, Kovacs C, Moreira P, Magnoni D, Saleh MH, Faintuch J.	Unsaturated Fatty Acids Improve Atherosclerosis Markers in Obese and Overweight Non-diabetic Elderly Patients.	Obes Surg. 2017 Oct;27(10):2663-2671. doi: 10.1007/s11695-017-2704-8. PMID: 28470492.
186.	Zhang, Y., Tang, W., Zhang, Y. *et al.*	Effects of integrated chronic care models on hypertension outcomes and spending: a multi-town clustered randomized trial in China.	*BMC Public Health* **17,** 244 (2017). https://doi.org/10.1186/s12889-017-4141-y
187.	Pasupathy S, Tavella R, Grover S, Raman B, Procter NEK, Du YT, Mahadavan G, Stafford I, Heresztyn T, Holmes A, Zeitz C, Arstall M, Selvanayagam J, Horowitz JD, Beltrame JF.	Early Use of N-acetylcysteine With Nitrate Therapy in Patients Undergoing Primary Percutaneous Coronary Intervention for ST-Segment-Elevation Myocardial Infarction Reduces Myocardial Infarct Size (the NACIAM Trial [N-acetylcysteine in Acute Myocardial Infarction]).	Circulation. 2017 Sep 5;136(10):894-903. doi: 10.1161/CIRCULATIONAHA.117.027575. Epub 2017 Jun 20. PMID: 28634219.
188.	Søndergaard L, Kasner SE, Rhodes JF, Andersen G, Iversen HK, Nielsen-Kudsk JE, Settergren M, Sjöstrand C, Roine RO, Hildick-Smith D, Spence JD, Thomassen L; Gore REDUCE Clinical Study Investigators.	Patent Foramen Ovale Closure or Antiplatelet Therapy for Cryptogenic Stroke..	N Engl J Med. 2017 Sep 14;377(11):1033-1042. doi: 10.1056/NEJMoa1707404. Erratum in: N Engl J Med. 2020 Mar 5;382(10):978. PMID: 28902580

189.	Clemenza F, Masson S, Conaldi PG, Di Carlo D, Boccanelli A, Mureddu GF, Gonzini L, Lucci D, Maggioni AP, Di Lenarda A, Nicolis EB, Vanasia M, Latini R;	AREA IN-CHF Investigators. Galectin-3 and the Mineralocorticoid Receptor Antagonist Canrenone in Mild Heart Failure.	Circ J. 2017 Sep 25;81(10):1543-1546. doi: 10.1253/circj.CJ-17-0656. Epub 2017 Aug 31. PMID: 28855452.
190.	Qin X, Li Y, Sun N, Wang H, Zhang Y, Wang J, Li J, Xu X, Liang M, Nie J, Wang B, Cheng X, Li N, Sun Y, Zhao L, Wang X, Hou FF, Huo Y.	Elevated Homocysteine Concentrations Decrease the Antihypertensive Effect of Angiotensin-Converting Enzyme Inhibitors in Hypertensive Patients.	Arterioscler Thromb Vasc Biol. 2017 Jan;37(1):166-172. doi: 10.1161/ATVBAHA.116.308515. Epub 2016 Nov 10. PMID: 27834686.
191.	Yang W, Zhou YJ, Fu Y, Qin J, Qin S, Chen XM, Guo JC, Wang Z, Zhan H, Li J, He JY, Hua Q.	Efficacy and Safety of Intravenous Urapidil for Older Hypertensive Patients with Acute Heart Failure: A Multicenter Randomized Controlled Trial.	Yonsei Med J. 2017 Jan;58(1):105-113. doi: 10.3349/ymj.2017.58.1.105. PMID: 27873502; PMCID: PMC5122625.
192.	Ellingsen Ø, Halle M, Conraads V, Støylen A, Dalen H, Delagardelle C, Larsen AI, Hole T, Mezzani A, Van Craenenbroeck EM, Videm V, Beckers P, Christle JW, Winzer E, Mangner N, Woitek F, Höllriegel	SMARTEX Heart Failure Study (Study of Myocardial Recovery After Exercise Training in Heart Failure) Group. High-Intensity Interval Training in Patients With Heart Failure With Reduced Ejection Fraction..	Circulation. 2017 Feb 28;135(9):839-849. doi: 10.1161/CIRCULATIONAHA.116.022924. Epub 2017 Jan 12. PMID: 28082387; PMCID: PMC5325251

	R, Pressler A, Monk-Hansen T, Snoer M, Feiereisen P, Valborgland T, Kjekshus J, Hambrecht R, Gielen S, Karlsen T, Prescott E, Linke A;		
193.	Williams B, Cockcroft JR, Kario K, Zappe DH, Brunel PC, Wang Q, Guo W.	Effects of Sacubitril/Valsartan Versus Olmesartan on Central Hemodynamics in the Elderly With Systolic Hypertension: The PARAMETER Study.	Hypertension. 2017 Mar;69(3):411-420. doi: 10.1161/HYPERTENSIONAHA.116.08556. Epub 2017 Jan 16. PMID: 28093466.
194.	Castro RRT, Porphirio G, Xavier SS, Moraes RS, Ferlin EL, Ribeiro JP, da Nóbrega ACL.	Cholinesterase inhibition reduces arrhythmias in asymptomatic Chagas disease.	Cardiovasc Ther. 2017 Oct;35(5). doi: 10.1111/1755-5922.12288. PMID: 28715142.
195.	Dominguez-Rodriguez A, Abreu-Gonzalez P, de la Torre-Hernandez JM, Gonzalez-Gonzalez J, Garcia-Camarero T, Consuegra-Sanchez L, Garcia-Saiz MD, Aldea-Perona A, Virgos-Aller T, Azpeitia A, Reiter RJ; MARIA Investigators.	Effect of intravenous and intracoronary melatonin as an adjunct to primary percutaneous coronary intervention for acute ST-elevation myocardial infarction: Results of the Melatonin Adjunct in the acute myocaRdial Infarction treated with Angioplasty trial.	J Pineal Res. 2017 Jan;62(1). doi: 10.1111/jpi.12374. Epub 2016 Nov 5. PMID: 27736028.
196.	Dalby AJ, Gottlieb S, Cyr DD,	Dual antiplatelet therapy in patients with diabetes and acute coronary syndromes	Am Heart J. 2017 Jun;188:156-166. doi:

	Magnus Ohman E, McGuire DK, Ruzyllo W, Bhatt DL, Wiviott SD, Winters KJ, Fox KAA, Armstrong PW, White HD, Prabhakaran D, Roe MT; TRILOGY ACS Investigators.	managed without revascularization.: 28577671.	10.1016/j.ahj.2017.03.015. Epub 2017 Mar 27. PMID
197.	Weisman D, Beinart R, Erez A, Koren-Morag N, Goldenberg I, Eldar M, Glikson M, Luria D.	Effect of supplemented intake of omega-3 fatty acids on arrhythmias in patients with ICD: fish oil therapy may reduce ventricular arrhythmia.	J Interv Card Electrophysiol. 2017 Sep;49(3):255-261. doi: 10.1007/s10840-017-0267-1. Epub 2017 Jun 29. PMID: 28664342.
198.	Eisen A, Ruff CT, Braunwald E, Hamershock RA, Lewis BS, Hassager C, Chao TF, Le Heuzey JY, Mercuri M, Rutman H, Antman EM, Giugliano RP.	Digoxin Use and Subsequent Clinical Outcomes in Patients With Atrial Fibrillation With or Without Heart Failure in the ENGAGE AF-TIMI 48 Trial..	J Am Heart Assoc. 2017 Jun 30;6(7):e006035. doi: 10.1161/JAHA.117.006035. PMID: 28666993; PMCID: PMC5586309
199.	Damorim IR, Santos TM, Barros GWP, Carvalho PRC.	Kinetics of Hypotension during 50 Sessions of Resistance and Aerobic Training in Hypertensive Patients: a Randomized Clinical Trial.	Arq Bras Cardiol. 2017 Apr;108(4):323-330. doi: 10.5935/abc.20170029. Epub 2017 Mar 30. PMID: 28380132; PMCID: PMC5421471.
200.	Hofmann R, James SK, Jernberg T, Lindahl B,	DETO2X–SWEDEHEART Investigators. Oxygen Therapy in Suspected Acute Myocardial Infarction..	N Engl J Med. 2017 Sep 28;377(13):1240-1249. doi:

	Erlinge D, Witt N, Arefalk G, Frick M, Alfredsson J, Nilsson L, Ravn-Fischer A, Omerovic E, Kellerth T, Sparv D, Ekelund U, Linder R, Ekström M, Lauermann J, Haaga U, Pernow J, Östlund O, Herlitz J, Svensson L;		10.1056/NEJMoa1706222. Epub 2017 Aug 28. PMID: 28844200
201.	Farha S, Saygin D, Park MM, Cheong HI, Asosingh K, Comhair SA, Stephens OR, Roach EC, Sharp J, Highland KB, DiFilippo FP, Neumann DR, Tang WHW, Erzurum SC.	Pulmonary arterial hypertension treatment with carvedilol for heart failure: a randomized controlled trial.	JCI Insight. 2017 Aug 17;2(16):e95240. doi: 10.1172/jci.insight.95240. PMID: 28814664; PMCID: PMC5621927.
202.	Gomes GB, Zazula AD, Shigueoka LS, Fedato RA, da Costa AB, Guarita-Souza LC, Baena CP, Olandoski M, Faria-Neto JR.	A Randomized Open-Label Trial to Assess the Effect of Plant Sterols Associated with Ezetimibe in Low-Density Lipoprotein Levels in Patients with Coronary Artery Disease on Statin Therapy.	J Med Food. 2017 Jan;20(1):30-36. doi: 10.1089/jmf.2016.0042. PMID: 28098515.
203.	Hougaard M, Hansen HS, Thayssen P, Antonsen L,	Influence of ezetimibe in addition to high-dose atorvastatin therapy on plaque composition in patients with ST-segment elevation myocardial infarction assessed by	Cardiovasc Revasc Med. 2017 Mar;18(2):110-117. doi: 10.1016/j.carrev.20

	Junker A, Veien K, Jensen LO.	serial: Intravascular ultrasound with iMap: the OCTIVUS trial.	16.11.010. Epub 2016 Nov 28. PMID: 27919638.
204.	Schwarz K, Singh S, Parasuraman SK, Rudd A, Shepstone L, Feelisch M, Minnion M, Ahmad S, Madhani M, Horowitz J, Dawson DK, Frenneaux MP.	Inorganic Nitrate in Angina Study: A Randomized Double-Blind Placebo-Controlled Trial..	J Am Heart Assoc. 2017 Sep 8;6(9):e006478. doi: 10.1161/JAHA.117.006478. PMID: 28887315; PMCID: PMC5634294
205.	Ambrosy AP, Cerbin LP, DeVore AD, Greene SJ, Kraus WE, O'Connor CM, Piña IL, Whellan DJ, Wojdyla D, Wu	A, Mentz RJ. Aerobic exercise training and general health status in ambulatory heart failure patients with a reduced ejection fraction-Findings from the Heart Failure and A Controlled Trial Investigating Outcomes of Exercise Training (HF-ACTION)trial.	Am Heart J. 2017 Apr;186:130-138. doi: 10.1016/j.ahj.2016.12.017. Epub 2017 Jan 19. PMID: 28454828.
206.	Link MS, Giugliano RP, Ruff CT, Scirica BM, Huikuri H, Oto A, Crompton AE, Murphy SA, Lanz H, Mercuri MF, Antman EM, Braunwald E; ENGAGE AF-TIMI 48 Investigators.	Stroke and Mortality Risk in Patients With Various Patterns of Atrial Fibrillation: Results From the ENGAGE AF-TIMI 48 Trial (Effective Anticoagulation With Factor Xa Next Generation in Atrial Fibrillation-Thrombolysis in Myocardial Infarction 48).	Circ Arrhythm Electrophysiol. 2017 Jan;10(1):e004267. doi: 10.1161/CIRCEP.116.004267. PMID: 28077507.
207.	Wright N, Wilson L, Smith M, Duncan B, McHugh P.	The BROAD study: A randomised controlled trial using a whole food plant-based diet in the community for obesity, ischaemic heart disease or diabetes.	Nutr Diabetes. 2017 Mar 20;7(3):e256. doi: 10.1038/nutd.2017.3. PMID:

			28319109; PMCID: PMC5380896.
208.	Barma M, Khan F, Price RJG, Donnan PT, Messow CM, Ford I, McConnachie A, Struthers AD, McMurdo MET, Witham MD.	Association between GDF-15 levels and changes in vascular and physical function in older patients with hypertension.	Aging Clin Exp Res. 2017 Oct;29(5):1055-1059. doi: 10.1007/s40520-016-0636-0. Epub 2016 Oct 12. PMID: 27734214; PMCID: PMC5589783.
209.	Kristensen SL, Mogensen UM, Jhund PS, Petrie MC, Preiss D, Win S, Køber L, McKelvie RS, Zile MR, Anand IS, Komajda M, Gottdiener JS, Carson PE, McMurray JJ.	Clinical and Echocardiographic Characteristics and Cardiovascular Outcomes According to Diabetes Status in Patients With Heart Failure and Preserved Ejection Fraction: A Report From the I-Preserve Trial (Irbesartan in Heart Failure With Preserved Ejection Fraction).	Circulation. 2017 Feb 21;135(8):724-735. doi: 10.1161/CIRCULATIONAHA.116.024593. Epub 2017 Jan 4. PMID: 28052977.
210.	Borer JS, Swedberg K, Komajda M, Ford I, Tavazzi L, Böhm M, Depre C, Wu Y, Maya J, Dominjon F.	Efficacy Profile of Ivabradine in Patients with Heart Failure plus Angina Pectoris.	Cardiology. 2017;136(2):138-144. doi: 10.1159/000449243. Epub 2016 Sep 10. PMID: 27614723.
211.	Lee HY, Kim SY, Choi KJ, Yoo BS, Cha DH, Jung HO, Ryu DR, Choi JH, Lee KJ, Park TH, Oh JH, Kim	Randomized, Multicenter, Double-blind, Placebo-controlled Study to Evaluate the Efficacy and the Tolerability of a Triple Combination of Amlodipine/Losartan/Rosuvastatin in Patients With Comorbid Essential Hypertension and Hyperlipidemia.	Clin Ther. 2017 Dec;39(12):2366-2379. doi: 10.1016/j.clinthera.2017.10.013. Epub 2017 Nov 14. PMID: 29150250.

	SM, Choi JY, Kim KH, Shim J, Kim WS, Choi SW, Park DG, Song PS, Hong TJ, Rhee MY, Rha SW, Park SW. A		
212.	Ahmadian M, Dabidi Roshan V, Ashourpore E.	Taurine Supplementation Improves Functional Capacity, Myocardial Oxygen Consumption, and Electrical Activity in Heart Failure	J Diet Suppl. 2017 Jul 4;14(4):422-432. doi: 10.1080/19390211.2016.1267059. Epub 2017 Jan 24. PMID: 28118062.
213.	Nammas W, Pietilä M, Romppanen H, Sia J, DeBelder A, Karjalainen PP.	Outcome of poor initial TIMI flow in patients presenting with acute coronary syndrome.	Scand Cardiovasc J. 2017 Oct;51(5):248-254. doi: 10.1080/14017431.2017.1346278. Epub 2017 Jun 30. PMID: 28666394.
214.	Wei He[1*], Gang Xu[1], Cheng Zhang[1], Lin Xu[2], Ting Li[3], Hua Yin[1], Hanqiong Zhang[1], Huawei Li[1], Xiaorong Hu[1], Hong Zhou[1] and Lisha Yang[1]	Clinical efficacy and safety of cardio-selective β-receptor blocker in management of AECOPD complicated with right heart failure	https://www.biomedres.info/biomedical-research/clinical-efficacy-and-safety-of-cardioselective-receptor-blocker-in-management-of-aecopd-complicated-with-right-heart-failure.html
215.	Fabris E, Kilic S, Schellings DAAM, Ten Berg JM, Kennedy MW, van Houwelingen KG, Giannitsis E, Kolkman E, Ottervanger JP, Hamm	Long-term mortality and prehospital tirofiban treatment in patients with ST elevation myocardial infarction.	Heart. 2017 Oct;103(19):1515-1520. doi: 10.1136/heartjnl-2017-311181. Epub 2017 Jul 5. PMID: 28679686.

	C, Van't Hof AWJ.		
216.	Rhee, MY., Ahn, T., Chang, K. *et al.*	The efficacy and safety of co-administration of fimasartan and rosuvastatin to patients with hypertension and dyslipidemia.	*BMC Pharmacol Toxicol* **18,** 2 (2017). https://doi.org/10.1186/s40360-016-0112-7
217.	Hanatani A, Shibata A, Kitada R, Iwata S, Matsumura Y, Doi A, Sugioka K, Takagi M, Yoshiyama M.	Administration of tolvaptan with reduction of loop diuretics ameliorates congestion with improving renal dysfunction in patients with congestive heart failure and renal dysfunction.	Heart Vessels. 2017 Mar;32(3):287-294. doi: 10.1007/s00380-016-0872-4. Epub 2016 Jul 6. PMID: 27385022.
218.	Chung MS, Yoon BI, Lee SH.	Clinical Efficacy and Safety of Naftopidil Treatment for Patients with Benign Prostatic Hyperplasia and Hypertension: A Prospective, Open-Label Study.	Yonsei Med J. 2017 Jul;58(4):800-806. doi: 10.3349/ymj.2017.58.4.800. PMID: 28540994; PMCID: PMC5447112.
219.	Mangiacapra F, Colaiori I, Ricottini E, Balducci F, Creta A, Demartini C, Minotti G, Di Sciascio G.	Heart Rate reduction by IVabradine for improvement of ENDothELial function in patients with coronary artery disease: the RIVENDEL study.	Clin Res Cardiol. 2017 Jan;106(1):69-75. doi: 10.1007/s00392-016-1024-7. Epub 2016 Aug 12. PMID: 27520989.
220.	Hwang YC, Yoon KH, Cha BS, Lee KW, Jang HC, Min KW, Chung CH, Lee MK.	Reduction in microalbuminuria by calcium channel blockers in patients with type 2 diabetes mellitus and hypertension-A randomized, open-label, active-controlled, superiority, parallel-group clinical trial.	Int J Clin Pract. 2017 Sep;71(9):e12987. doi: 10.1111/ijcp.12987. Epub 2017 Aug 24. PMID: 28840637; PMCID: PMC5637912.
221.	Hegde SM, Claggett B, Shah AM, Lewis EF, Anand I, Shah SJ, Sweitzer NK, Fang JC, Pitt B, Pfeffer MA, Solomon SD.	Physical Activity and Prognosis in the TOPCAT Trial (Treatment of Preserved Cardiac Function Heart Failure With an Aldosterone Antagonist).	Circulation. 2017 Sep 12;136(11):982-992. doi: 10.1161/CIRCULATIONAHA.117.028002. Epub 2017 Jun 21. PMID: 28637881; PMCID: PMC6592611.

222.	Gaggin HK, Truong QA, Gandhi PU, Motiwala SR, Belcher AM, Weiner RB, Baggish AL, Januzzi JL Jr.	Systematic Evaluation of Endothelin 1 Measurement Relative to Traditional and Modern Biomarkers for Clinical Assessment and Prognosis in Patients With Chronic Systolic Heart Failure: Serial Measurement and Multimarker Testing.	Am J Clin Pathol. 2017 May 1;147(5):461-472. doi: 10.1093/ajcp/aqx014. PMID: 28398455.
223.	Miyoshi T, Ejiri K, Kohno K, Nakahama M, Doi M, Munemasa M, Murakami M, Takaishi A, Kawai Y, Sato T, Sato K, Oka T, Takahashi N, Sakuragi S, Mima A, Enko K, Hosogi S, Nanba S, Hirami R, Nakamura K, Ito H;	RINC Study Collaborators. Effect of remote ischemia or nicorandil on myocardial injury following percutaneous coronary intervention in patients with stable coronary artery disease: A randomized controlled trial..	Int J Cardiol. 2017 Jun 1;236:36-42. doi: 10.1016/j.ijcard.2017.02.028. Epub 2017 Feb 10. PMID: 28214082
224.	Fernandes AC, McIntyre T, Coelho R, Prata J, Maciel MJ.	Brief psychological intervention in phase I of cardiac rehabilitation after acute coronary syndrome.	Rev Port Cardiol. 2017 Sep;36(9):641-649. English, Portuguese. doi: 10.1016/j.repc.2017.01.005. Epub 2017 Sep 4. PMID: 28882655.
225.	Suojanen L, Haring A, Tikkakoski A, Koskela JK, Tahvanainen AM, Huhtala H, Kähönen M, Sipilä K, Eräranta A, Mustonen JT, Kivistö K, Pörsti IH.	Haemodynamic Influences of Bisoprolol in Hypertensive Middle-Aged Men: A Double-Blind, Randomized, Placebo-Controlled Cross-Over Study.	Basic Clin Pharmacol Toxicol. 2017 Aug;121(2):130-137. doi: 10.1111/bcpt.12771. Epub 2017 May 3. PMID: 28256104.

226.	Komajda M, Isnard R, Cohen-Solal A, Metra M, Pieske B, Ponikowski P, Voors AA, Dominjon F, Henon-Goburdhun C, Pannaux M, Böhm M;	prEserveD left ventricular ejectIon fraction chronic heart Failure with ivabradine studY (EDIFY) Investigators. Effect of ivabradine in patients with heart failure with preserved ejection fraction: the EDIFY randomized placebo-controlled trial.	Eur J Heart Fail. 2017 Nov;19(11):1495-1503. doi: 10.1002/ejhf.876. Epub 2017 Apr 30. PMID: 28462519.
227.	Cunha AR, D'El-Rei J, Medeiros F, Umbelino B, Oigman W, Touyz RM, Neves MF.	Oral magnesium supplementation improves endothelial function and attenuates subclinical atherosclerosis in thiazide-treated hypertensive women.	J Hypertens. 2017 Jan;35(1):89-97. doi: 10.1097/HJH.0000000000001129. PMID: 27759579.
228.	Ulrich S, Hasler ED, Saxer S, Furian M, Müller-Mottet S, Keusch S, Bloch KE.	Effect of breathing oxygen-enriched air on exercise performance in patients with precapillary pulmonary hypertension: randomized, sham-controlled cross-over trial.	Eur Heart J. 2017 Apr 14;38(15):1159-1168. doi: 10.1093/eurheartj/ehx099. PMID: 28329240.
229.	Botto GL, Padeletti L, Covino G, Pieragnoli P, Liccardo M, Mariconti B, Favale S, Molon G, De Filippo P, Bolognese L, Landolina M, Raciti G, Boriani G.	Electrical treatment of atrial arrhythmias in heart failure patients implanted with a dual defibrillator CRT device. Results from the TRADE-HF study.	Int J Cardiol. 2017 Jun 1;236:181-186. doi: 10.1016/j.ijcard.2017.01.101. Epub 2017 Jan 19. PMID: 28131706.
230.	Nedogoda SV, Stojanov VJ.	Single-Pill Combination of Perindopril/Indapamide/Amlodipine in Patients with Uncontrolled Hypertension: A Randomized Controlled Trial.	Cardiol Ther. 2017 Jun;6(1):91-104. doi: 10.1007/s40119-017-0085-7. Epub 2017 Feb 8. PMID: 28181192; PMCID: PMC5446818.
231.	Sharifi, M. H.,	Effects of a therapeutic lifestyle change diet and supplementation with Q10 plus L-	*Journal of cardiovascular and*

	Eftekhari, M. H., Ostovan, M. A., & Rezaianazadeh, A. (2017).	carnitine on quality of life in patients with myocardial infarction: A randomized clinical trial.	*thoracic research*, *9*(1), 21–28. https://doi.org/10.15171/jcvtr.2017.03
232.	Holte E, Kleveland O, Ueland T, Kunszt G, Bratlie M, Broch K, Michelsen AE, Bendz B, Amundsen BH, Aakhus S, Damås JK, Gullestad L, Aukrust P, Wiseth R.	Effect of interleukin-6 inhibition on coronary microvascular and endothelial function in myocardial infarction..	Heart. 2017 Oct;103(19):1521-1527. doi: 10.1136/heartjnl-2016-310875. Epub 2017 Apr 21. PMID: 28432157
233.	Jeppesen AN, Hvas AM, Grejs AM, Duez C, Ilkjær S, Kirkegaard H.	Platelet aggregation during targeted temperature management after out-of-hospital cardiac arrest: A randomised clinical trial.	Platelets. 2018 Jul;29(5):504-511. doi: 10.1080/09537104.2017.1336213. Epub 2017 Jul 31. PMID: 28758873.
234.	Ozyıldız AG, Eroglu S, Bal U, Atar I, Okyay K, Muderrisoglu H.	Effects of Carvedilol Compared to Nebivolol on Insulin Resistance and Lipid Profile in Patients With Essential Hypertension.	J Cardiovasc Pharmacol Ther. 2017 Jan;22(1):65-70. doi: 10.1177/1074248416644987. Epub 2016 Jul 7. PMID: 27093951.
235.	González-Saiz L, Fiuza-Luces C, Sanchis-Gomar F, Santos-Lozano A, Quezada-Loaiza CA, Flox-Camacho A, Munguía-Izquierdo D, Ara I, Santalla A, Morán M, Sanz-Ayan P,	Benefits of skeletal-muscle exercise training in pulmonary arterial hypertension: The WHOLEi+12 trial.	Int J Cardiol. 2017 Mar 15;231:277-283. doi: 10.1016/j.ijcard.2016.12.026. PMID: 28189191.

	Escribano-Subías P, Lucia A.		
236.	Ertelt K, Brener SJ, Mehran R, Ben-Yehuda O, McAndrew T, Stone GW.	Comparison of Outcomes and Prognosis of Patients With Versus Without Newly Diagnosed Diabetes Mellitus After Primary Percutaneous Coronary Intervention for ST-Elevation Myocardial Infarction (the HORIZONS-AMI Study).	Am J Cardiol. 2017 Jun 15;119(12):1917-1923. doi: 10.1016/j.amjcard.2017.03.016. Epub 2017 Mar 29. PMID: 28427734.
237.	Gwag HB, Kim EK, Park TK, Lee JM, Yang JH, Song YB, Choi JH, Choi SH, Lee SH, Chang SA, Park SJ, Lee SC, Park SW, Jang WJ, Lee M, Chun WJ, Oh JH, Park YH, Choe YH, Gwon HC, Hahn JY.	Cardioprotective Effects of Intracoronary Morphine in ST-Segment Elevation Myocardial Infarction Patients Undergoing Primary Percutaneous Coronary Intervention: A Prospective, Randomized Trial.	J Am Heart Assoc. 2017 Apr 3;6(4):e005426. doi: 10.1161/JAHA.116.005426. PMID: 28373244; PMCID: PMC5533032.
238.	Ibrahim NE, Januzzi JL, Rabideau DJ, Gandhi PU, Gaggin HK.	Serial Heart Rates, Guideline-Directed Beta Blocker Use, and Outcomes in Patients With Chronic Heart Failure With Reduced Ejection Fraction	Am J Cardiol. 2017 Sep 1;120(5):803-808. doi: 10.1016/j.amjcard.2017.05.052. Epub 2017 Jun 15. PMID: 28728744.
239.	Kadoglou NP, Mandila C, Karavidas A, Farmakis D, Matzaraki V, Varounis C, Arapi S, Perpinia A, Parissis J.	Effect of functional electrical stimulation on cardiovascular outcomes in patients with chronic heart failure.	Eur J Prev Cardiol. 2017 May;24(8):833-839. doi: 10.1177/2047487316687428. Epub 2017 Jan 12. PMID: 28079427.
240.	Seferovic JP, Claggett B, Seidelmann SB, Seely EW, Packer M, Zile MR,	Effect of sacubitril/valsartan versus enalapril on glycaemic control in patients with heart failure and diabetes: a post-hoc analysis from the PARADIGM-HF trial.	Lancet Diabetes Endocrinol. 2017 May;5(5):333-340. doi: 10.1016/S2213-8587(17)30087-6. Epub 2017 Mar 18.

	Rouleau JL, Swedberg K, Lefkowitz M, Shi VC, Desai AS, McMurray JJV, Solomon SD.		PMID: 28330649; PMCID: PMC5534167.
241.	Abdelaziz HK, Elkilany W, Khalid S, Sabet S, Saad M.	Efficacy and safety of intracoronary verapamil versus sodium nitroprusside for the prevention of microvascular obstruction during primary percutaneous coronary intervention for ST-segment elevation myocardial infarction.	Coron Artery Dis. 2017 Jan;28(1):11-16. doi: 10.1097/MCA.0000000000000423. PMID: 27556348.
242.	Givertz MM, Stevenson LW, Costanzo MR, Bourge RC, Bauman JG, Ginn G, Abraham WT;	CHAMPION Trial Investigators. Pulmonary Artery Pressure-Guided Management of Patients With Heart Failure and Reduced Ejection Fraction.	J Am Coll Cardiol. 2017 Oct 10;70(15):1875-1886. doi: 10.1016/j.jacc.2017.08.010. PMID: 28982501.
243.	Liu Z, Hao H, Yin C, Chu Y, Li J, Xu D.	Therapeutic effects of atorvastatin and ezetimibe compared with double-dose atorvastatin in very elderly patients with acute coronary syndrome.	Oncotarget. 2017 Jun 20;8(25):41582-41589. doi: 10.18632/oncotarget.15078. PMID: 28177908; PMCID: PMC5522285.
244.	Shi C, Men L, Yu C, Yao J, Bai R, Yang Y, Sun L, Sun G, Song G, Zhang Y, Xing Q, Du J.	Atherosclerosis associated with dynamic inflammation changes after multifactorial intervention in short-duration type 2 diabetes: A randomized, controlled, 10-year follow-up trial.	J Diabetes Complications. 2017 Aug;31(8):1286-1292. doi: 10.1016/j.jdiacomp.2017.05.008. Epub 2017 May 25. PMID: 28610945.
245.	Fors A, Swedberg K, Ulin K, Wolf A, Ekman I.	Effects of person-centred care after an event of acute coronary syndrome: Two-year follow-up of a randomised controlled trial.	Int J Cardiol. 2017 Dec 15;249:42-47. doi: 10.1016/j.ijcard.2017.08.069. Epub 2017 Sep 6. PMID: 28893432.
246.	Desai AS, Vardeny O, Claggett B, McMurray JJ, Packer	Reduced Risk of Hyperkalemia During Treatment of Heart Failure With Mineralocorticoid Receptor Antagonists by Use of Sacubitril/Valsartan Compared With	JAMA Cardiol. 2017 Jan 1;2(1):79-85. doi: 10.1001/jamacardio

	M, Swedberg K, Rouleau JL, Zile MR, Lefkowitz M, Shi V, Solomon SD.	Enalapril: A Secondary Analysis of the PARADIGM-HF Trial.	.2016.4733. PMID: 27842179.
247.	Kalizki T, Schmidt BMW, Raff U, Reinold A, Schwarz TK, Schneider MP, Schmieder RE, Schneider A.	Low dose-eplerenone treatment decreases aortic stiffness in patients with resistant hypertension.	J Clin Hypertens (Greenwich). 2017 Jul;19(7):669-676. doi: 10.1111/jch.12986. Epub 2017 Feb 17. PMID: 28211216; PMCID: PMC8031041.
248.	Zeymer U, Werdan K, Schuler G, Zahn R, Neumann FJ, Fürnau G, de Waha S, Schneider S, Thiele H. Editor's Choice-	Impact of immediate multivessel percutaneous coronary intervention versus culprit lesion intervention on 1-year outcome in patients with acute myocardial infarction complicated by cardiogenic shock: Results of the randomised IABP-SHOCK II trial.	Eur Heart J Acute Cardiovasc Care. 2017 Oct;6(7):601-609. doi: 10.1177/2048872616668977. Epub 2016 Sep 21. PMID: 27655918.
249.	Butler J, Epstein SE, Greene SJ, Quyyumi AA, Sikora S, Kim RJ, Anderson AS, Wilcox JE, Tankovich NI, Lipinski MJ, Ko YA, Margulies KB, Cole RT, Skopicki HA, Gheorghiade M.	Intravenous Allogeneic Mesenchymal Stem Cells for Nonischemic Cardiomyopathy: Safety and Efficacy Results of a Phase II-A Randomized Trial.	Circ Res. 2017 Jan 20;120(2):332-340. doi: 10.1161/CIRCRESAHA.116.309717. Epub 2016 Nov 16. PMID: 27856497.
250.	Storniolo CE, Casillas R, Bulló M, Castañer O, Ros E, Sáez	A Mediterranean diet supplemented with extra virgin olive oil or nuts improves endothelial markers involved in blood pressure control in hypertensive women.	Eur J Nutr. 2017 Feb;56(1):89-97. doi: 10.1007/s00394-015-1060-5. Epub

	GT, Toledo E, Estruch R, Ruiz-Gutiérrez V, Fitó M, Martínez-González MA, Salas-Salvadó J, Mitjavila MT, Moreno JJ.		2015 Oct 8. PMID: 26450601.
